# Fungal Energy Channelling Sustains Soil Animal Communities Across Forest Types and Regions

**DOI:** 10.1111/ele.70122

**Published:** 2025-05-08

**Authors:** André Junggebauer, Melissa Jüds, Bernhard Klarner, Jens Dyckmans, Melanie M. Pollierer, Stefan Scheu

**Affiliations:** ^1^ Department of Animal Ecology J.‐F. Blumenbach Institute of Zoology and Anthropology, University of Göttingen Göttingen Germany; ^2^ Centre for Stable Isotope Research and Analysis University of Göttingen Göttingen Germany; ^3^ Centre of Biodiversity and Sustainable Land Use University of Göttingen Göttingen Germany

**Keywords:** basal resources, Biodiversity Exploratories, central Europe, CSIA, energy channels, energy flux, forest management, soil invertebrates, trophic positions

## Abstract

Emerging evidence suggests that microbivory prevails in soil animal communities, yet the relative importance of bacteria, fungi and plants as basal resource energy channels across taxa and forest types remains unstudied. We developed a novel framework combining stable isotope analysis of essential amino acids (eAAs) and energy fluxes to quantify basal resource contributions and trophic positions of meso‐ and macrofauna detritivores (Collembola, Oribatida, Diplopoda, Isopoda, Lumbricidae) and predators (Mesostigmata, Chilopoda) in 48 forest sites of different management intensity across Germany. Fungal energy channelling dominated, with the highest energy fluxes and 73% fungal eAAs across forests and regions. Chilopoda, however, acquired more energy from bacteria and plants. Energy fluxes to Lumbricidae were highest, but decreased, alongside those to other macrofauna, in acidic forests. Trophic positions varied between regions, reflecting changes in community structure linked to regional factors. Our findings highlight the stability and pivotal role of fungal energy channelling for forest soil animal communities.

## Graphical abstract

We developed a novel framework combining stable isotope analysis of essential amino acids and energy fluxes to quantify basal resource contributions and trophic positions of meso‐ and macrofauna detritivores (Collembola, Oribatida, Diplopoda, Isopoda, Lumbricidae) and predators (Mesostigmata, Chilopoda) in 48 forest sites of different management intensity across Germany. Fungal energy channelling dominated, with the highest energy fluxes and 73% fungal eAAs across forests and regions, highlighting the stability and pivotal role of the fungal energy channel in sustaining forest soil animal communities.

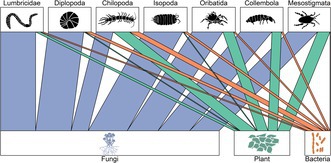

## 1 Introduction

Trophic interactions in soil form an essential component of terrestrial ecosystems by recycling dead organic matter (Scheu and Setälä 2002). This is largely attributed to the activity of fungi and bacteria colonising dead plant material and animal remains (Crowther et al. 2019; Zeng et al. 2024). However, forest soil communities of mesofauna (< 2 mm) and macrofauna (> 2 mm body width) facilitate decomposition by grazing on microbes and by feeding on the ‘detrital complex’, i.e., plant material colonised by fungi and bacteria (Schaefer 1990; Steffan et al. 2017). Although omnivory is generally high in soil animal communities, detritivores use bacteria, fungi and plants as basal resources to different degrees, resulting in compartmentalisation of forest soil food webs with multiple parallel energy channels (Moore et al. 2005; Pollierer et al. 2009). Disentangling the importance of basal resource energy channels and their persistence under human influence provides critical insights into the stability of soil animal communities and the provisioning of below‐ground ecosystem services (Manlick and Newsome 2022; Moore and Hunt 1988).

Natural beech forests in central Europe have largely been replaced by production forests and are now managed on a long‐term basis (Fischer et al. 2010; McGrath et al. 2015). Management intensification in Europe is among the main drivers of aboveground arthropod biodiversity loss (Seibold et al. 2019; van Klink et al. 2024), while soil animal communities have been shown to be rather resistant to management practices and more influenced by regional differences in climate, pH and soil type (Junggebauer et al. 2024; Pollierer and Scheu 2017; Pollierer et al. 2021). However, tree harvesting and the prevalence of immature and/or non‐native coniferous trees in managed forests alter abiotic and biotic conditions, reducing fungal biomass and diversity (Goldmann et al. 2015; Richter et al. 2018). Generally, disturbed and highly productive systems favour the bacterial energy pathway, while fungi tend to dominate in near‐natural systems (Bardgett and Cook 1998; Moore et al. 2005; Wardle et al. 2004). Furthermore, recalcitrant spruce needles with high C/N ratios may reduce the importance of the plant energy channel, as litter quality is an important factor in bottom‐up regulated soil food webs (Ott et al. 2014). However, the main energy pathways in soil food webs and their stability across forest types of different management intensities and regions are poorly understood (Ferlian and Scheu 2014; Pollierer et al. 2015).

Compound‐specific isotope analysis (CSIA) of carbon and nitrogen in amino acids (AA) has recently emerged as a powerful tool to provide detailed insights into trophic niches of soil animals (Pollierer et al. 2019). Specifically, stable isotope ratios of ^13^C/^12^C (*δ* ^13^C) in essential amino acids (eAAs) synthesised by bacteria, fungi and plants can be distinguished, due to lineage‐specific enzymatic pathways (Larsen et al. 2009, 2013, 2022). Most animals cannot synthesise eAAs de novo at sufficient rates and must obtain them from dietary proteins, resulting in little to no isotopic fractionation of eAAs across trophic levels (Fox et al. 2019). This allows tracing of *δ* ^13^C values in eAAs of consumers back to their basal resource and thus to quantify the relative importance of bacteria, fungi and plants for channelling carbon in soil animal food webs (Larsen et al. 2009). Additionally, trophic positions (TPs) can be estimated from natural variations in nitrogen isotope ratios of ^15^N/^14^N (*δ* ^15^N), in source and trophic amino acids, such as phenylalanine and glutamic acid. With each trophic transfer, *δ* ^15^N values of glutamic acid consistently increase, while the *δ* ^15^N values of phenylalanine change little, thereby reflecting the isotopic composition of the basal resources, regardless of spatiotemporal changes (Chikaraishi et al. 2011; Ohkouchi et al. 2017). Furthermore, ^15^N discrimination is similar between microbes and animals, allowing for the integration of intermediate microbial trophic levels into TP estimates (Steffan et al. 2015).

Studies employing CSIA for soil macrofauna identified plants as a major basal resource for certain earthworm species (Potapov et al. 2019; Zhong et al. 2024), whereas Li et al. (2022) and Lux et al. (2024) demonstrated the importance of fungi for the diet of mesofauna taxa, such as Collembola and Oribatida at the community level. However, a holistic approach including multiple animal groups is needed to elucidate the trophic structure and energy channelling, and their drivers in soil food webs, as interactions of multiple meso‐ and macrofauna species contribute to the functioning of soil systems (Barnes et al. 2018; Potapov 2022). Moreover, no study has yet integrated process rates such as energy fluxes into this context, although the relative contributions of essential amino acids to consumer diets have frequently been conceptualised as energy channels (Besser et al. 2023; Elliott Smith et al. 2021). This is surprising, as energy fluxes are closely tied to multiple ecosystem functions and processes, including decomposition (Barnes et al. 2018; Moore and de Ruiter 2012). Consequently, linking the relative contributions of basal resources quantified by CSIA to species abundance, biomass, metabolic activity and resource assimilation efficiencies in an energy flux framework provides a holistic perspective on the primary energy channels sustaining soil animal communities.

Here, we used community‐level compound‐specific isotope analysis of amino acids (CSIA‐AA) coupled with energy flux estimates for a broad range of major meso‐ and macrofauna detritivores (Collembola, Oribatida, Diplopoda, Isopoda Lumbricidae) and predators (Mesostigmata, Chilopoda) of forests of different management intensity in three regions of Germany to investigate three hypotheses:

1. Fungi are the dominant basal resource, with this being more pronounced in detritivorous mesofauna (Collembola, Oribatida) than in detritivorous macrofauna (Diplopoda, Isopoda, Lumbricidae).
2. The relative importance of the bacterial energy channel increases in managed forests, but this varies between regions, reflecting previously observed differences in soil animal community compositions due to regional factors such as pH, soil type and climate.
3. Trophic positions of meso‐ and macrofauna increase in extensively managed coniferous forests due to the decreasing importance of the plant energy channel and a shift towards the bacterial energy channel.

## 2 Material and Methods

### 2.1 Study Sites

The study was carried out in the experimental forest sites of the ‘Biodiversity Exploratories’ (www.biodiversity‐exploratories.de; Fischer et al. 2010), located across three regions of Germany i.e., northern, central and southern Germany. Forest sites in northern Germany (elevation 3–140 m a.s.l., precipitation 500–600 mm, pH 3.5) are in the Schorfheide‐Chorin and are characterised by sandy Podzols. Forests in central Germany (elevation 285–550 m a.s.l., annual precipitation 500–800 mm, pH 5.2) are in the Hainich‐Dün, with clay‐rich Luvisols, Cambisols and Stagnosols developed on limestone. Forests in southern Germany (elevation 460–860 m a.s.l., annual precipitation 700–1000 mm, pH 5.3) are in the Swabian Alb, with Cambisols and Leptosols. Forests in the three regions comprise age‐class beech forests ( *Fagus sylvatica* ) and coniferous forests with Norway spruce ( *Picea abies* ) in central and southern regions and Scots pine ( *Pinus sylvestris* ) in the north. In each of the three regions, forests differ in the proportion of non‐native tree species, stand age and stand density due to harvesting and thinning (Schall and Ammer 2013, 2023), resulting in a management gradient ranging from near‐natural beech forests, that have been unmanaged for at least 60 years (tree age 150 years; Beech150), to managed mature beech forests (tree age 70 years; Beech70), to managed young beech forests (tree age 30 years; Beech30) and to coniferous forests (approximate tree age of 70 years; Coniferous; Figure S1). Each forest type is replicated four times in each region, resulting in a total of 48 study sites.

### 2.2 Sampling of Soil Animals and Leaf Litter

Two soil cores, one 20 cm and one 5 cm in diameter, were collected in 2020 at random locations in a 5 m × 5 m subplot at each of the 48 sites (Table S1). Soil animals were extracted from litter and the 5 cm below using high‐gradient heat extractors (Kempson et al. 1963; Macfadyen 1961). From the larger soil cores, we collected detritivores, i.e., millipedes (Diplopoda), oribatid mites (Oribatida), springtails (Collembola), woodlice (Isopoda), and predators, i.e., centipedes (Chilopoda) and mesostigmatid mites (Mesostigmata), for CSIA‐AA. Earthworms (Lumbricidae) were collected by hand for 30 min, using mustard as an expellant (Eisenhauer et al. 2008), applying 10 L of a 10% mustard solution to 0.25 m^2^ of forest soil. All specimens were measured under a Stemi 508 dissecting microscope equipped with an Axiocam ERc 5 s (Zeiss, Jena, Germany) and determined to species level whenever possible, using the keys of Sims and Gerard (1985) for Lumbricidae, Andersson et al. (2006) and Klausnitzer (2019) for Chilopoda and Diplopoda and Schaefer (2018) for Isopoda. For Oribatida, Collembola and Mesostigmata, we randomly selected up to 250 individuals to obtain a representative subcommunity with biomass suitable for CSIA‐AA. To obtain complete community data, all individuals present in the smaller soil cores were counted, measured and determined to species level using the keys of Weigmann (2006) for Oribatida, Hopkin (2007) for Collembola and (Karg 1989, 1993) for Mesostigmata. Community data for Oribatida have been published elsewhere (Junggebauer et al. 2024). Note that body sizes for Collembola were taken from Potapov et al. (2023), while for Oribatida, we measured the length of all juveniles and used the length of adults from Weigmann (2006). Fresh leaf litter was collected from leaf traps at all forest sites in autumn 2019. All animal and litter samples were lyophilised, homogenised with a pestle and stored in a desiccator for further analysis. In total, we extracted amino acids from 344 samples (Table S2).

### 2.3 Amino Acid Extraction and Analysis

About 0.5–5 mg of dried and homogenised animal tissue and 15 mg of leaf litter were hydrolysed and derivatised, alongside standard mixtures of pure AAs with known *δ* ^13^C and *δ* ^15^N values after batches of approximately 20 samples, following Larsen et al. (2016; Supporting Information S1: Protocol S1). AAs of samples and standards were measured in triplicates using a Thermo Trace GC 1310 gas chromatograph linked to a delta V mass spectrometer (Thermo, Bremen, Germany) equipped with an Agilent J&W VF‐35 ms GC column (30 m × 0.32 mm × 1.00 μm). Derivatised standards were measured after every 3 to 5 samples to ensure reproducibility of measurements (Table S3). Nitrogen isotope ratios of AAs were expressed relative to atmospheric N by normalising measured values (vs. reference gas) using scales derived from the known *δ* ^15^N values (versus atmospheric N) in AAs of the derivatised standards. Carbon isotope ratios were corrected for added carbon during derivatisation following O'Brien et al. (2002) and expressed relative to Vienna Pee Dee Belemnite. We report all following stable isotope data using the *δ* notation (‰). Reliable isotopic values for carbon were obtained for Ala, Asx, Glu, Ile*, Leu*, Phe*, Pro, Ser, Thr* and Val* (asterisks denoting eAAs used for the analysis). The total number of chromatograms used in the analysis was 318 for carbon and 297 for nitrogen.

### 2.4 Statistical Analysis

All statistical analyses were performed in R v 4.4.3 (R Core Team 2025). To trace the biosynthetic origin of eAAs from bacteria, fungi and plants in soil animal communities, we used stable isotope fingerprints, *sensu* Larsen et al. (2009), using linear discriminant analysis (*MASS*::lda; Venables and Ripley 2002). We trained the model with *δ* ^13^C eAA values from bacteria, fungi and plants from Larsen et al. (2013, 2016), Pollierer et al. (2020) and our own leaf litter, resulting in three distinct clusters (Figure S2). Next, we classified consumers to basal resources, based on their proximity to the centroids of basal resources, and used manova to test for significant differences in linear discriminant means between animal groups (7 levels: Lumbricidae, Diplopoda, Chilopoda, Isopoda, Oribatida, Collembola, Mesostigmata), forest type (4 levels: Beech150, Beech70, Beech30, Coniferous) and their interaction with region (3 levels: northern Germany, central Germany, southern Germany). We then tested differences in linear discriminant means between forest types and regions (including their interaction) in separate manovas for each animal group. To quantify the proportion of eAAs from bacteria, fungi and plants in soil animal groups of different forest types and regions, we ran Bayesian mixing models (chain length = 10,000; burn‐in = 5000) implemented in *MixSIAR* (Stock et al. 2018) with mean‐centred *δ* ^13^C eAA values. Model convergence was confirmed using the Gelman‐Rubin diagnostic, which was below 1.05 for all variables (Phillips et al. 2014). The interaction of forest type and region in mixing models was excluded due to lack of convergence. Quantified contribution of eAAs to consumers was illustrated with the *bipartite* package (Dormann et al. 2009) and used to derive energy fluxes from basal resources to animal groups (see Box 1).

TP of soil animal groups was calculated following Chikaraishi et al. (2011):

(2) $$\mathrm{TP}=1+\left[\frac{\;\delta^{15}N_{\mathrm{Glu}}-\delta^{15}N_{\mathrm{Phe}}+\beta }{{\mathrm{TDF}}_{\mathrm{Glu}-\mathrm{Phe}}}\right]$$

where *δ* ^15^N_*Glu*_ and *δ* ^15^N_Phe_ are the *δ* ^15^N values in Glu and Phe of soil animals, *β* the difference between *δ* ^15^N values in Glu and Phe of the primary producer, i.e., 8.77 ± 1.17 for beech leaves and 7.84 ± 1.00 for spruce needles collected from our study sites, and TDF_Glu−Phe_ the universally used trophic discrimination factor of 7.60‰ ± 1.20‰ (Chikaraishi et al. 2014). Differences in TP were assessed with linear models using the same model structure as above. Normality of response variables in all models was checked with the *fitdistrplus* package (Delignette‐Muller and Dutang 2015), log‐transformed if necessary, and subjected to residual diagnostics using *DHARMa* (Hartig 2024). Pairwise contrasts were calculated with the Tukey test implemented in *emmeans* (Lenth 2024).

BOX 1 **Calculating energy fluxes from basal resources to animal groups.**

Calculation of energy fluxes generally followed Jochum et al. (2021) but was modified to integrate data from CSIA‐AA. First, body length and width were used in group‐specific biomass regressions to calculate fresh animal body weight (Table S4). Fresh weight of all individuals of the respective animal group was multiplied by their density (ind. m^−2^) at each site and summed to derive group‐specific community biomasses per square metre for all 48 sites. Then, for each site, metabolic rates of soil animal communities were calculated with taxon‐specific regressions from Ehnes et al. (2011), mean temperatures measured at 10 cm above the ground in our study regions in 2020 (northern Germany 10.3°C, central Germany 9.3°C, southern Germany 8.6°C; Wöllauer et al. 2021) and the summed biomass on a square metre to derive the metabolic rate per square metre. Resource assimilation efficiencies (e_a_) were calculated based on the nitrogen (N) concentration (%) of bacteria, fungi and plants, respectively, according to the linear model formula proposed by Jochum et al. (2017):

(1) $$\;\text{logit}\;e_{a}=0.471\times \text{foodN}\;\left(\%\right)-2.097$$

and temperature corrected using the mean temperature across regions (9.4°C) following Lang et al. (2017). Nitrogen concentrations of saprotrophic and ectomycorrhizal fungi were taken from the meta‐analysis of Zhang and Elser (2017), resulting in a mean nitrogen concentration of 3.14% ± 1.67% (*n* = 58) and an assimilation efficiency of 0.30 for fungi. The nitrogen concentration of plants was derived from leaf litter collected from the Biodiversity Exploratories sites in 2021 and measured using a coupled system of an elemental analyser and a mass spectrometer (Reineking et al. 1993). The assimilation efficiency for plants was 0.15, based on a mean nitrogen content of 1.30% ± 0.18% (*n* = 26, including conifer needles and beech leaves). For the bacterial nitrogen concentration, we averaged the lower and upper ranges of the reported C/N ratios for bacteria and soil microbial biomass in Chapin et al. (2011), Chen et al. (2023) and Reiners (1986), resulting in a nitrogen concentration of 7.07% ± 1.62% and an assimilation efficiency of 0.73. Bacterial nitrogen concentrations were converted from C/N ratios by assuming that carbon makes up 50% of bacterial dry mass (Whitman et al. 1998). Assimilation efficiencies for bacteria, fungi and plants were divided by the metabolic rate of each animal group to calculate the energy inputs. Finally, to derive energy fluxes (J h^−1^) from each basal resource, we multiplied the energy input by the relative contribution of bacteria, fungi and plants estimated for all animal groups in forest types and regions, respectively, using Bayesian mixing models (see above). Because predators (Chilopoda and Mesostigmata) acquire energy from basal resources through prey rather than directly feeding on them, we further multiplied the energy fluxes of each energy channel by the assimilation efficiency of carnivores (0.91; Lang et al. 2017). To test whether energy fluxes from basal resources in soil animal groups differ between forest types and regions, we constructed two linear models, i.e. one with animal group, basal resource (3 levels: bacteria, fungi, plants) and forest type as factors, and one with animal group, basal resource and region as factors. We then built separate linear models for all animal groups that included basal resource and forest type or basal resource and region and their interactions as factors.

## 3 Results

### 3.1 Energy Channels of Forest Soil Animal Communities

**FIGURE 1:**
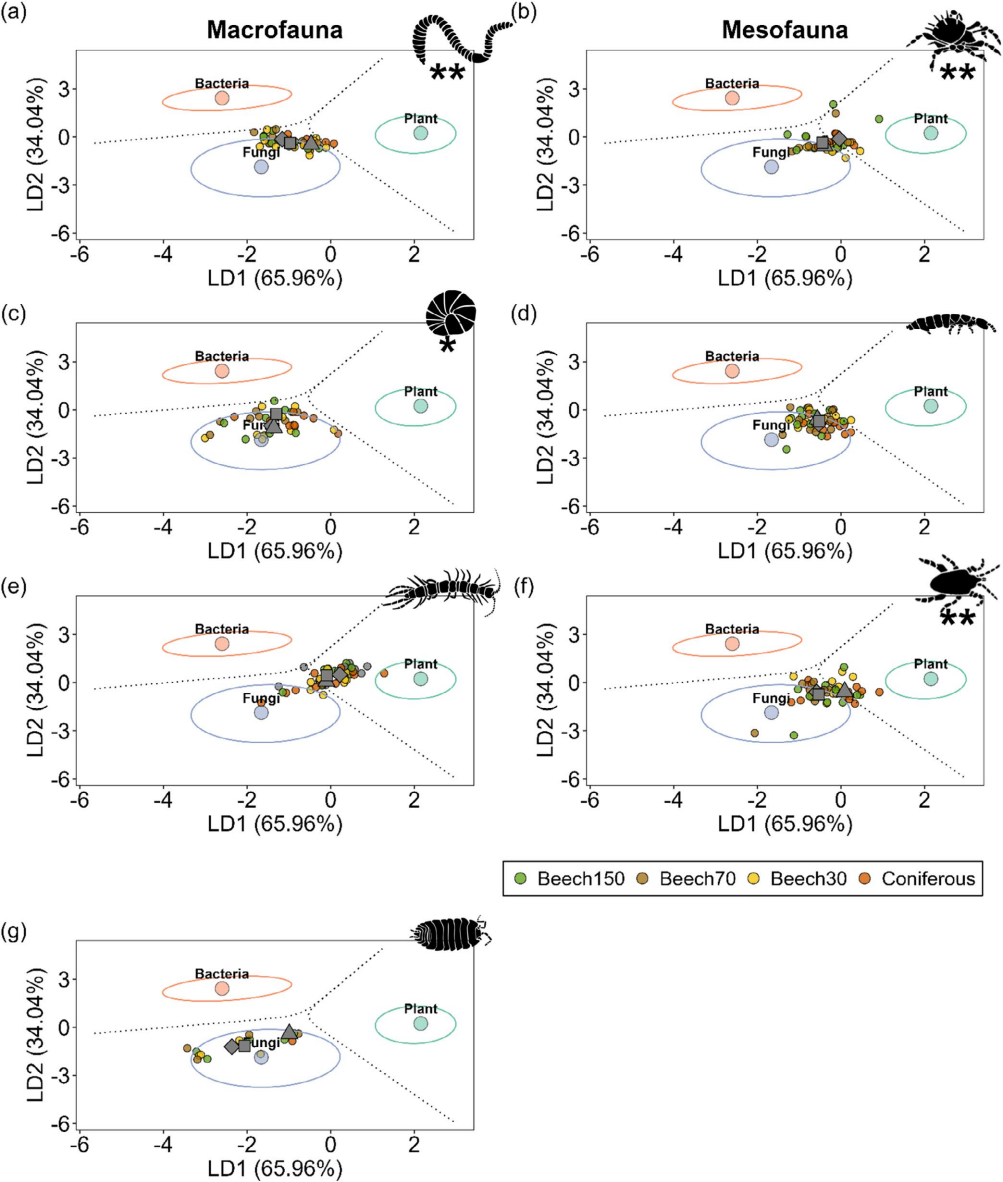
Linear discriminant analysis (LDA) of *δ* ^13^C values in eAAs for soil animal communities [Lumbricidae (a), Oribatida (b), Diplopoda (c), Collembola (d), Chilopoda (e), Mesostigmata (f) and Isopoda (g)] in unmanaged beech forests (Beech150), old managed beech forests (Beech70), young managed beech forests (Beech30) and coniferous forests (Coniferous). Linear discriminant means for the three regions are shown as grey squares (southern Germany), diamonds (central Germany) and triangles (northern Germany). Asterisks denote significant differences in linear discriminant means between regions as indicated by manova. Training data used as end members to classify *δ* ^13^C values in eAAs of consumers to their biosynthetic origin were taken from plants (light green, *n* = 59), fungi (light blue, *n* = 39) and bacteria (light orange, *n* = 26). Ellipses around linear discriminant means of basal resources (points) represent 75% confidence intervals.

Out of a total of 276 soil animal communities, LDA classified 209 (75.7%) communities to fungi, 65 (23.5%) to plants and two communities (0.7%) to bacteria. Linear discriminant means differed significantly between forest types, with soil animal communities in coniferous forests clustering closer to plants than those in beech forests (Figure S3, Table S5). However, manova for individual groups indicated that the effect of forest type only affected the *δ* ^13^C eAA values of Oribatida, and this depended on region (Table S5). Linear discriminant means of *δ* ^13^C values also differed significantly between groups and the interaction between group and region (Figure 1, Table S5).

**FIGURE 2:**
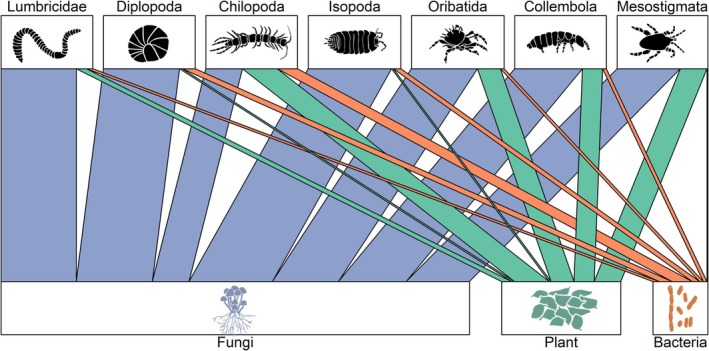
Energy channelling from basal resources [fungi (blue), plants (green) and bacteria (orange)] to soil animal groups. The width of the energy channels represents the mean relative proportion of essential amino acids from basal resources (in %) as indicated by Bayesian mixing models. The size of the boxes of basal resources is scaled to their relative importance across all animal groups, adding up to 100%. Note that the underlying model contained only animal group as factor, as the relative proportions of eAAs in animal groups between regions and forest types were within 95% credible intervals (Figure S4b,c).

**FIGURE 3:**
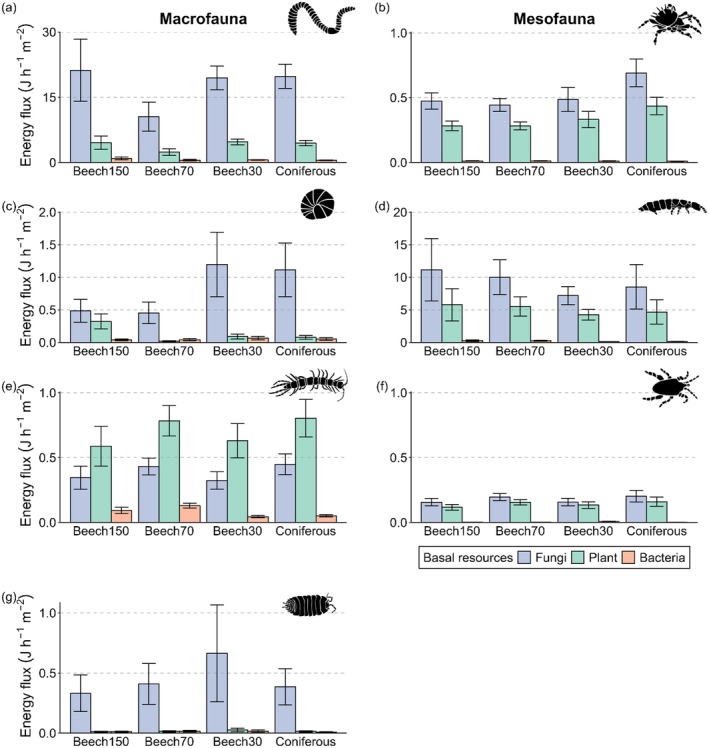
Mean energy fluxes (J h^−1^ m^−2^ ± SE) from basal resources of fungi (blue), plants (green) and bacteria (light orange) to soil animal groups [Lumbricidae (a), Oribatida (b), Diplopoda (c), Collembola (d), Chilopoda (e), Mesostigmata (f), Isopoda (g)] in unmanaged beech forests (Beech150), old managed beech forests (Beech70), young managed beech forests (Beech30) and coniferous forests (Coniferous). Energy fluxes were calculated using specific assimilation efficiencies as a function of the N content of basal resources and the relative contribution of essential amino acids from fungi, plants and bacteria to animal groups in different forest types as indicated by Bayesian mixing models. Note different scales of *y*‐axes.

**FIGURE 4:**
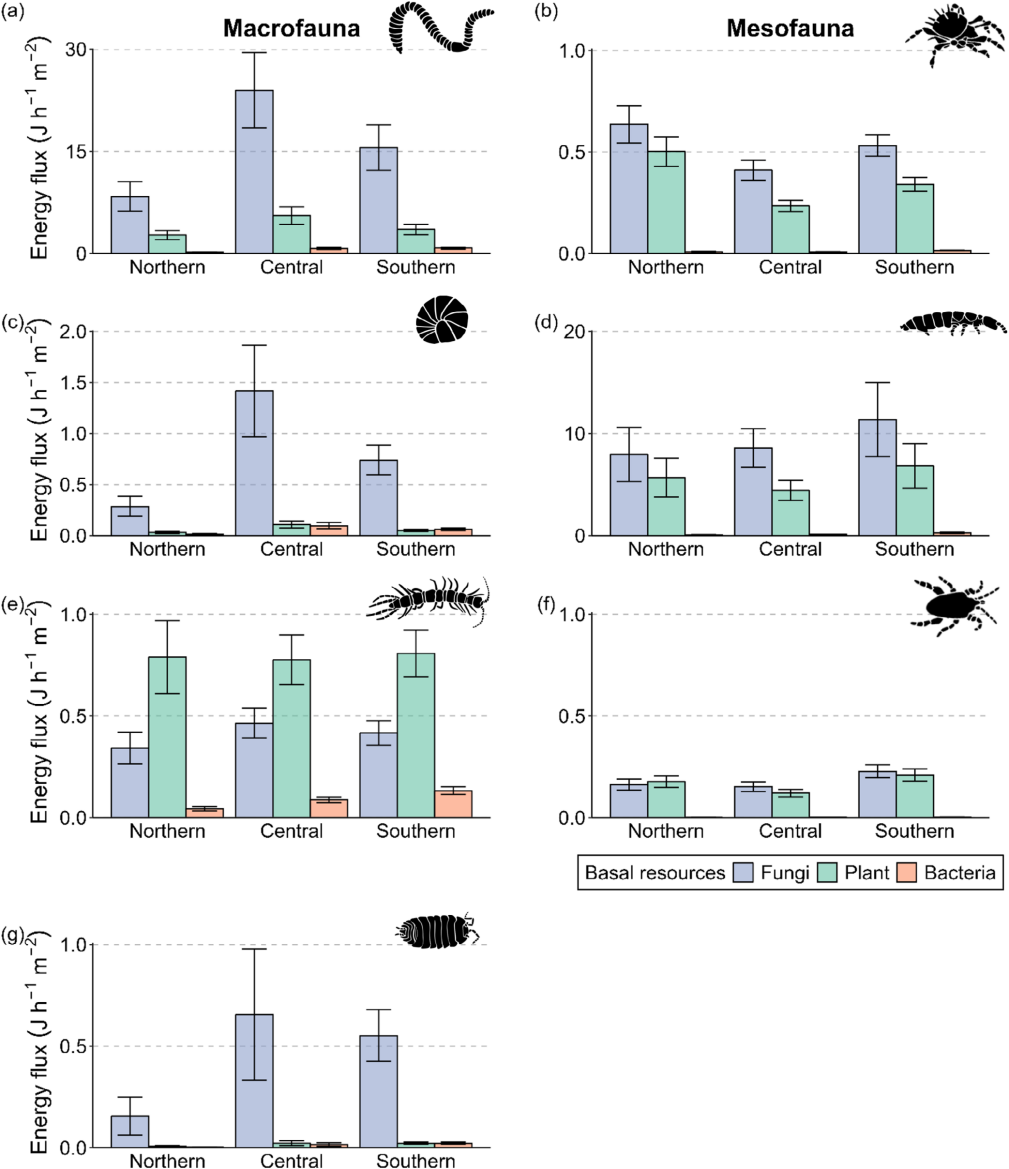
Mean energy fluxes (J h^−1^ m^−2^ ± SE) from basal resources of fungi (blue), plants (green) and bacteria (light orange) to animal groups [Lumbricidae (a), Oribatida (b), Diplopoda (c), Collembola (d), Chilopoda (e), Mesostigmata (f), Isopoda (g)] in northern Germany (Northern), central Germany (Central) and southern Germany (Southern). Energy fluxes were calculated using specific assimilation efficiencies as a function of the N content of basal resources and the relative contribution of essential amino acids from fungi, plants and bacteria to animal groups in different regions as indicated by Bayesian mixing models. Note different scales of *y*‐axes.

Fingerprints of Lumbricidae and Mesostigmata clustered close to fungi, although they shifted significantly towards plants in northern Germany (Figure 1a,f). By contrast, *δ* ^13^C values of Oribatida communities were significantly closer to plants in central Germany (Figure 1b). Diplopoda and Isopoda were uniformly assigned to fungi, although in the former, the linear discriminant means for communities of southern Germany differed significantly from those of the two other regions (Figure 1c,g). Collembola communities were located close to fungi, whereas Chilopoda communities were located between bacteria, fungi and plants (Figure 1d,e). Mixing models indicated that fungal eAAs had the highest relative contribution to basal resources with 73.0% ± 16.3% (mean ± SD), followed by plants with 18.5% ± 13.7% and bacteria with 8.5% ± 7.1% (Figure 2). However, fungal‐derived eAAs contributed only 39.9% ± 3.2% to the diet of Chilopoda, which was significantly lower than in all other soil animal groups (Figure 2, Figure S4a). Contributions of fungi to basal resources in the other soil animal groups ranged from 69.1% ± 3.3% in Mesostigmata to 90.7% ± 3.8% in Isopoda, with the latter being significantly higher than in Oribatida, Collembola and Mesostigmata (Figure 2, Figure S4a). By contrast, Chilopoda had the highest relative contribution of eAAs from plants (38.2% ± 2.7%) and bacteria (21.9% ± 2.1%), with the latter being significantly higher than in all other soil animal groups except Diplopoda (Figure S4a). Relative proportions of eAAs between soil animal communities of different forest types and regions were within 95% credible intervals (Figure S4b,c). Similarly, relative energy fluxes from fungi, plants and bacteria remained similar for all soil animal groups except Mesostigmata across forest types and, except for Oribatida, across regions (no significant interaction between Forest type × Basal resource or × Region × Basal resource; Table S6). However, energy fluxes differed significantly between animal groups, resources and their interaction (Table S6). Although energy fluxes from fungi dominated in soil animal communities across forest types and regions, centipedes acquired more energy from plants than from fungi (Figure 3e). The total flux (summed fluxes from fungi, plants and bacteria) was highest for Lumbricidae, with 22.4 ± 19.3 J h^−1^ m^−2^ (mean ± SD), which was more than one order of magnitude higher than that of all other soil animal communities except Collembola (14.4 ± 17.3 J h^−1^ m^−2^; Figure S5). Linear models indicated that energy fluxes were similar between forest types but differed for animal groups within forest types (Figure 3, Table S6). Specifically, energy fluxes to Lumbricidae were significantly lower in old managed beech forests than in the other forests studied (Figure 3a). Energy fluxes differed significantly between regions for all animal groups, with lower fluxes in northern Germany for macrofauna (Figure 4a,c,e,g, Table S6). Fluxes to Oribatida were lower in central Germany, while the energy flux from plants increased in northern Germany. By contrast, fluxes to Collembola and Mesostigmata were highest in southern Germany (Figure 4b,d,f).

### 3.2 Trophic Positions of Forest Soil Animal Communities

**FIGURE 5:**
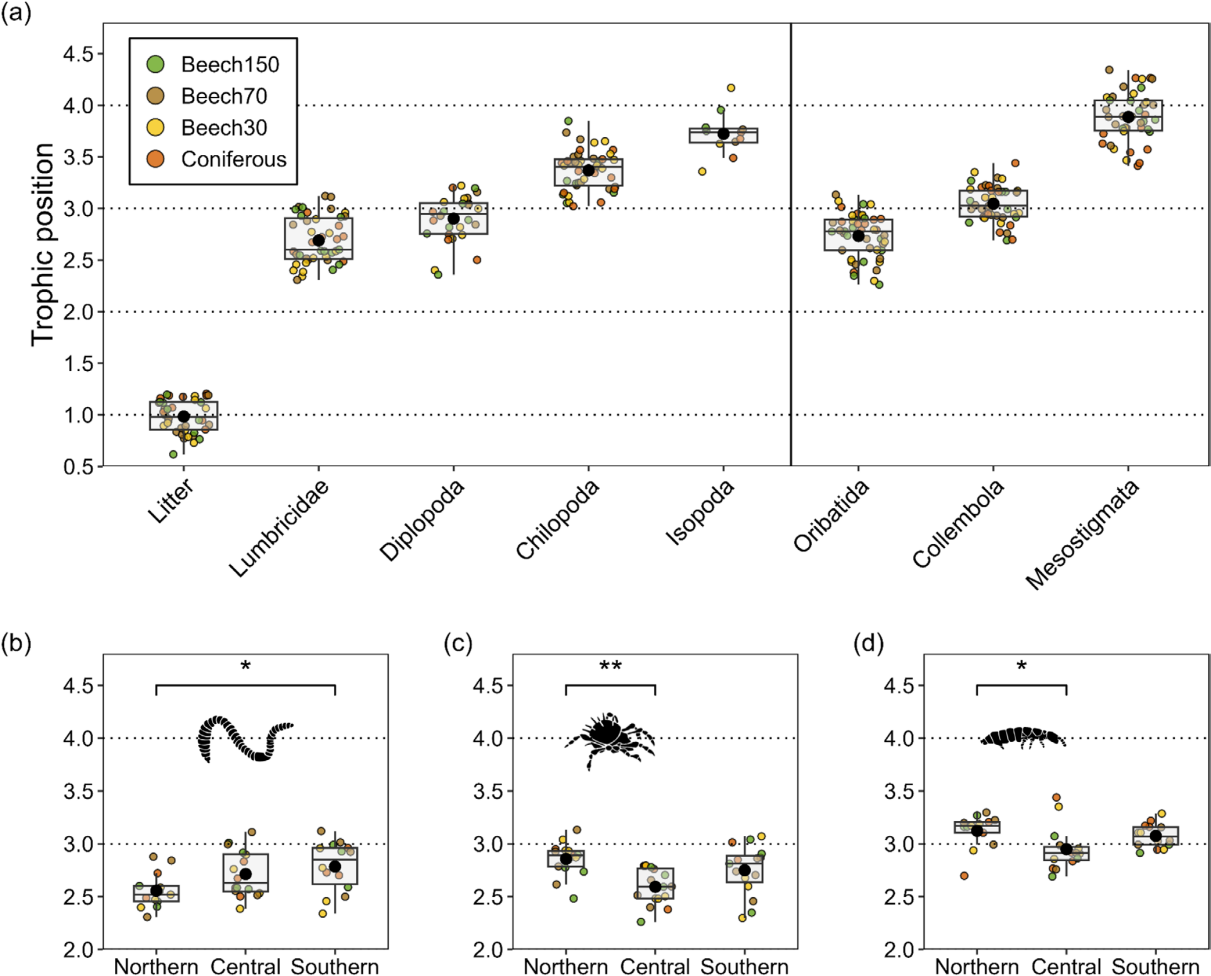
Trophic position of litter and animal groups across three study regions in Germany in unmanaged beech forests (Beech150), old managed beech forests (Beech70), young managed beech forests (Beech30) and coniferous forests (Coniferous) calculated from *δ* ^15^N values of glutamic acid and phenylalanine (a), and of Lumbricidae (b), Oribatida (c) and Collembola (d) across forest types in northern, central and southern Germany; black circles represent means, horizontal lines medians and boxes the interquartile range. The vertical line in (a) separates litter and macrofauna taxa from mesofauna taxa. Trophic position 2 represents consumers feeding on plant resources such as litter, trophic position 3 indicates feeding on fungi, bacteria or animals of trophic position 2, and trophic position 4 represents predators of microbivores and intraguild predators.

TPs differed significantly between soil animal groups, regions and their interaction (Table S5). All detritivores occupied significantly higher TPs compared to litter, which had a TP of 0.98 ± 0.15; mean ± SD. Among detritivores, TPs were lowest in Lumbricidae (2.69 ± 0.22) and increased to Oribatida (2.73 ± 0.21), Diplopoda (2.90 ± 0.23), Collembola (3.05 ± 0.17) and Isopoda (3.72 ± 0.21) (Figure 5a).

Predators mostly occupied higher TPs than detritivores, with a mean TP of 3.37 ± 0.19 for Chilopoda and 3.89 ± 0.24 for Mesostigmata. Across soil animal groups, TPs ranged from a minimum of 2.26 for Oribatida in an unmanaged beech forest to a maximum of 4.34 for Mesostigmata in an old managed beech forest. However, TPs varied little with forest type (Figure 5a, Figure S6) but differed significantly between regions in Lumbricidae, Oribatida and Collembola (Figure 5b–d, Table S5). TPs of Lumbricidae in northern Germany (2.55 ± 0.17) were significantly lower than in southern Germany 2.78 ± 0.23. By contrast, the TPs of Oribatida and Collembola were highest in northern Germany, with 2.85 ± 0.15 and 3.12 ± 0.16, respectively, both significantly higher than in central Germany.

## 4 Discussion

We used community‐level CSIA‐AA coupled with energy fluxes and showed that fungal energy channelling dominates in soil animal communities of temperate beech and coniferous forests. Energy channels differed only between animal groups, with centipedes receiving less eAAs from fungi and higher energy fluxes from plants. Energy fluxes to Lumbricidae exceeded those to all other animal groups except Collembola by an order of magnitude but decreased in young managed beech forests. Energy fluxes were similar between forest types in all other animal groups but decreased to macrofauna groups in acidic forests of our sites in northern Germany. Similarly, trophic positions of Lumbricidae, Oribatida and Collembola at our sites in northern Germany differed from those in the other two regions studied but varied little with forest type for all animal groups.

### 4.1 Energy Channels and Trophic Positions of Detritivores

In contrast to our first hypothesis, Lumbricidae predominantly obtained eAAs from fungi, followed by plants and, to a lesser extent, from bacteria. This contrasts with previous studies conducted in grasslands and arable fields, reporting that earthworms acquired most of their eAAs from bacteria (Larsen et al. 2016; Zhong et al. 2024). However, forests support significantly higher fungal biomass than grasslands and arable fields (Jackson et al. 2019), which likely explains the high contribution of fungi to the diet of Lumbricidae and their rather high mean TP of 2.69. Similar to our results, previous studies have shown that trophic niches of Lumbricidae are conserved across forest types but vary between species of different ecological groups (epigeic, endogeic, anecic), which have different feeding strategies (Ferlian and Scheu 2014; Potapov et al. 2019). Endogeic species consume large amounts of mineral soil with microbially processed organic matter and thus occupy higher trophic positions (Hyodo et al. 2012), whereas epigeic species ingest more plant material resulting in lower trophic positions (Potapov et al. 2019). Therefore, plant‐associated fingerprints and lower trophic positions of Lumbricidae in northern sites result from communities dominated by epigeic earthworms, as the acidic sandy soils (pH ~3.5) in this region are unsuitable for endogeic and anecic species (Pollierer et al. 2021). The lack of endogeic and anecic species also results in lower community biomass at our northern sites and thus lower energy flux to Lumbricidae, contrasting with the central and southern sites (Figure S7). However, total energy flux to Lumbricidae exceeded that to all other soil animal taxa and this conforms to previous studies showing that earthworms are the dominant contributors to food web energy fluxes in forest ecosystems (Potapov et al. 2024; Schaefer 1990).

Trophic niches of the dominant soil microarthropods, Collembola and Oribatida, were similar, although fungal contribution to eAAs and trophic positions of Collembola were higher. Trophic positions of both animal groups were similar across forest types but were significantly higher at our sites in northern than those in central Germany, likely mainly reflecting differences in community composition. Relative abundances of Oppiidae, presumably predominantly living as scavengers and predators of nematodes, are significantly higher at our northern sites where they contribute substantially to the biomass of Oribatida (Junggebauer et al. 2024; Maraun et al. 2023). Collembola communities at our northern sites also differ from those of the two other sites, but information on differences in feeding strategies is lacking (Pollierer and Scheu 2017). However, Li et al. (2022) found that root‐trenching significantly decreased the trophic position of Collembola in forests at sites similar to ours in northern Germany, suggesting that rhizosphere microorganisms but also animal prey such as nematodes form an important component of their diet. Presumably, high root density in organic layers of sandy forest soils, such as those at our northern sites, increases the availability of rhizosphere prey to Collembola (Richter et al. 2023). Higher trophic positions of Oribatida and Collembola thus point to longer trophic chains due to increased consumption of microorganisms and/or nematodes in the rhizosphere (Li et al. 2022; Steffan et al. 2015). Notably, total energy flux to Collembola was at least five times higher compared to all other animal groups except Lumbricidae. Collembola have high metabolic rates, rapid life cycles and high fecundity, each contributing to increased overall energy demand (Hopkin 1997; Meehan 2006). Following earthworms, they also had the second highest biomass, contributing to their high community metabolism (Brown et al. 2004; Ehnes et al. 2011; Figure S8) and consequently, the high energy fluxes observed in our and previous studies (Potapov et al. 2023, 2024). Energy fluxes to Oribatida were significantly lower at our central than at northern and southern sites, which likely is associated with high densities of endogeic and anecic earthworms, which alter the structure of organic layers and reduce available niche space for oribatid mites due to their burrowing activity (Eisenhauer 2010). Finally, the high energy flux from plants to Oribatida at our northern sites also points to feeding on root‐associated nematodes, potentially predominantly root‐feeding ones.

Diplopoda and Isopoda had the highest relative proportions of fungal eAAs among soil animals, with 83% and 91%, respectively. This, together with the predominantly fungal‐based eAAs in Lumbricidae, disproves our first hypothesis that fungi are less important as a basal resource energy channel in macrofauna than in mesofauna detritivores. Similarly, fungal‐based energy fluxes dominated in both groups, whereas the amount of energy channelled from plants and bacteria was negligible. Notably, overall fluxes were similar across forest types but lower at our northern than at our central and southern study sites, reflecting lower biomass of Diplopoda and Isopoda in acidic forest soils (Stašiov et al. 2021). Previous studies have found Diplopoda to include both litter‐ and microbial‐feeding species (Scheu and Falca 2000). However, our results suggest that species with microbial diets predominantly contribute to the biomass of Diplopoda communities in temperate forests. Fingerprints of Diplopoda from our southern study sites clustered significantly closer to bacteria than in the other two regions, suggesting different dietary preferences among species or varying gut microbial contributions (Nweze et al. 2024; Semenyuk and Tiunov 2011). The particularly high TP of 3.7 for Isopoda is likely explained by coprophagy. Feeding on faeces is common in Isopoda and allows them to recycle microbial proteins rich in eAAs, mainly from fungi, which were the primary energy channel (Gunnarsson and Tunlid 1986; Zimmer 2002).

### 4.2 Energy Channels and Trophic Positions of Predators

The trophic position and energy channels of Chilopoda were similar between forest types and regions, although the relative proportions of eAAs from fungi and bacteria differed significantly between Chilopoda and each of the detritivore taxa studied. Similarly, the relative energy flux from plants was higher than that from fungi, which contrasts with all other animal groups studied, suggesting that Chilopoda in temperate forests predominantly prey on soil animals not included here. Macrofauna detritivores, such as Diplopoda and Isopoda, were previously assumed to be of little importance for invertebrate predators, as they are well protected by large body size, strong sclerotization and chemical deterrents (Scheu 2002). Similar defence mechanisms apply to Oribatida, at least for adults, making them unattractive prey for Chilopoda (Eitzinger et al. 2018). However, in contrast to previous studies, our results do not indicate a close association with Collembola or Lumbricidae as prey (Günther et al. 2014; Hickerson et al. 2005). This may be partially explained by the fact that we analysed total Chilopoda without separating Geophilomorpha and Lithobiomorpha, which may differ in prey spectrum (Eitzinger et al. 2018). Lithobiomorpha have been suggested to be more closely associated with the fungal energy channel by feeding predominantly on Collembola, whereas Geophilomorpha feed more heavily on bacteria‐ and plant‐feeding prey, such as enchytraeids and anecic earthworms (Ferlian and Scheu 2014; Ferlian et al. 2012; Poser 1988). Indeed, enchytraeids have recently been shown to rely heavily on eAAs from plants and bacteria and may form an important prey group for centipede communities in temperate forests (Larsen et al. 2016). To further improve the resolution of trophic positions and energy channels, future studies using community‐level CSIA, both below‐ and aboveground, may focus on ecological rather than large taxonomic groups. Not separating ecological groups of Chilopoda, Lumbricidae (see above) and Collembola (Potapov et al. 2016), differing in feeding strategies and body size in our study, may have masked the hypothesised differences in energy channels and trophic positions between forest types by averaging the variability within groups.

Mesostigmata occupied the highest trophic position in the soil animal communities studied (Klarner et al. 2013), which was significantly higher than that of Chilopoda but similar to that of Isopoda. Trophic position, relative proportion of eAAs and energy fluxes suggest that Mesostigmata feed predominantly on Collembola and Oribatida. We found no evidence that Mesostigmata form a substantial fraction of the prey of Chilopoda (Eitzinger et al. 2018). Feeding on Collembola is well known for certain species of Mesostigmata (Klarner et al. 2013; Walter and Proctor 1998) and our results indicate that this generally applies to Mesostigmata communities in temperate beech and spruce forests. Our results also suggest that Mesostigmata may prey on oribatid mites, most likely on less protected juveniles, which has previously only been reported under laboratory conditions (Schneider and Maraun 2005; Walter and Proctor 1998). Mesostigmata have a broad food spectrum (Crotty and Adl 2019), which is also suggested by the shift towards the plant energy channel at our northern study sites as indicated by fingerprinting. As emphasised above, rhizosphere prey may be more important at our northern sites than in the other two regions. Mesostigmata prey heavily on Collembola but potentially also on predatory nematodes feeding on root‐associated nematodes, but this needs further investigation.

### 4.3 Effects of Forest Type and Region on Soil Animal Trophic Niches

Energy channels and trophic positions of soil animal groups varied mostly between regions and were little affected by forest type/management. This partly contradicts our second and third hypotheses and previous studies on microbial food webs, which found a higher importance of the bacterial energy channel in disturbed and more productive ecosystems (Moore et al. 2005). Regional differences between our study areas, particularly between northern Germany and the other two regions, include different soil types, pH and climate, and our results indicate that these differences are more important for soil animal food webs and communities than differences in forest type and management intensity (Manlick et al. 2024; Pollierer et al. 2021). Consistent channelling of basal resources across regions, despite differences in the composition of animal groups, supports previous assumptions of high functional redundancy in soil food webs, which contributes to the stability of below‐ground communities (Magilton et al. 2019; Pollierer et al. 2021; Scheu and Setälä 2002). Finally, the variability observed using *δ* ^13^C fingerprints is likely to be less precise in reflecting different proportions of basal resources in consumer diets, as linear discriminant means do not allow accurate reconstruction of mixed diets compared to mixing models (Besser et al. 2023; Manlick and Newsome 2022).

## 5 Conclusions

We presented a novel approach integrating CSIA data into an energy flux framework to quantify energy channels and trophic positions of soil animal detritivores and predators in temperate forests. Our results highlight consistent fungal energy channelling across forest types and regions, suggesting that management and regional soil properties little affect the basal resources used by soil animal communities. Channelling of bacterial and plant basal resources was highest in Chilopoda, probably due to preying on Enchytraeidae, which were not included in our study. Energy fluxes were generally highest in Lumbricidae and Collembola and lower across macrofauna taxa in acidic forests at our northern sites compared to central and southern sites. Forest type only affected the energy flux to Lumbricidae, which was lower in young managed beech forests. Trophic positions were similar between forest types, but higher for Collembola and Oribatida at our northern sites, suggesting feeding on rhizosphere prey. By contrast, the lower trophic positions of Lumbricidae reflected the dominance of litter‐dwelling species in acidic sandy soils. Despite regional variation in energy fluxes and trophic positions, likely associated with changing community compositions, the consistent energy channelling of basal resources across forest types and regions indicates high functional redundancy and stability of forest soil food webs of the temperate zone.

## Author Contributions

S.S. designed the study. A.J., M.J., B.K. and M.M.P. collected, measured and identified the soil animals. A.J. and J.D. extracted and measured amino acids. Final data were analysed by A.J. and M.M.P., A.J. led the writing of the manuscript, and all authors contributed to drafts and approved the final version of the manuscript.

## Conflicts of Interest

The authors declare no conflicts of interest.

### Peer Review

The peer review history for this article is available at https://www.webofscience.com/api/gateway/wos/peer‐review/10.1111/ele.70122.

## Supporting information


Data S1.

## Data Availability

All data and code necessary to reproduce the results of this study are publicly available in Dryad (https://doi.org/10.5061/dryad.2ngf1vj03) and Zenodo (https://doi.org/10.5281/zenodo.14186786).
