# Supplementary material for: Fungal Energy Channelling Sustains Soil Animal Communities Across Forest Types and Regions
Source: Ecol Lett. 2025 May 8;28(5):e70122. doi: 10.1111/ele.70122 (PMC12061547; doi:10.1111/ele.70122)
Supplement: Supplementary file 1 — Data S1. [file ELE-28-0-s001.docx]

Fungal energy channeling sustains soil animal communities across forest types and regions

André Junggebauer, Melissa Jüds, Bernhard Klarner, Jens Dyckmans, Melanie M. Pollierer, Stefan Scheu

Ecology Letters

**Protocol S1.**

About 0.5 – 5 mg of dry animal tissue and approximately 15 mg of dried leaf litter were weighed into Pyrex culture tubes, which were flushed with N_2_ and sealed. Tubes were placed in a heating block to hydrolyze tissue in 1 ml of 6 N HCl at 110°C for 20 h. To remove lipophilic compounds, we added 2 ml of hexane/dichloromethane to each sample, rinsed the tubes with N_2_ and sealed them before vortexing for 30 s. The aqueous phase was then filtered through a sterilized Pasteur pipette lined with glass wool, to remove any solids. Samples were transferred into 4 ml dram vials and 50 µl nor-leucine (5 µmol ml^-1^) was added as an internal standard before being evaporated to dryness under a stream of N_2_ at 110°C in a heating block for 30 min. Dried samples were then stored at -18°C until derivatization following the procedure of Corr et al. (2007). In short, we methylated the samples using an acidified methanol solution and dissolved the methylated AAs in a mixture of acetic anhydride, triethylamine and acetone (1:2:5) to obtain N-acetyl methyl ester derivates. To minimize the oxidation of AAs, derivatization, methylation and acetylation were conducted under continuous N_2_ flow. To account for added carbon during derivatization, we derivatized a standard mixture of pure AAs with known δ^13^C and δ^15^N values along with batches of approximately 20 samples.

Table S1: Geographic locations of the 48 studied forest sites, the main tree species and average pH from two measurements taken in 2017 at 0-10 cm soil depth (see Schöning *et al.* 2024).

| EP_Plot_ID | Region | Latitude | Longitude | Main_Tree_Species | pH_Mean |
| --- | --- | --- | --- | --- | --- |
| AEW01 | Southern | 48.48 | 9.33 | *Picea abies* | 3.34 |
| AEW02 | Southern | 48.38 | 9.35 | *Picea abies* | 4.84 |
| AEW03 | Southern | 48.41 | 9.36 | *Picea abies* | 5.63 |
| AEW04 | Southern | 48.4 | 9.24 | *Fagus sylvatica* | 6.76 |
| AEW05 | Southern | 48.42 | 9.41 | *Fagus sylvatica* | 4.45 |
| AEW06 | Southern | 48.39 | 9.45 | *Fagus sylvatica* | 5.59 |
| AEW07 | Southern | 48.4 | 9.26 | *Fagus sylvatica* | 5.01 |
| AEW08 | Southern | 48.38 | 9.38 | *Fagus sylvatica* | 6.43 |
| AEW09 | Southern | 48.37 | 9.42 | *Fagus sylvatica* | 6.12 |
| AEW11 | Southern | 48.49 | 9.32 | *Picea abies* | 3.42 |
| AEW17 | Southern | 48.4 | 9.24 | *Fagus sylvatica* | 6.53 |
| AEW18 | Southern | 48.37 | 9.23 | *Fagus sylvatica* | 4.69 |
| AEW25 | Southern | 48.48 | 9.42 | *Fagus sylvatica* | 5.13 |
| AEW27 | Southern | 48.4 | 9.47 | *Fagus sylvatica* | 4.58 |
| AEW30 | Southern | 48.37 | 9.37 | *Fagus sylvatica* | 5.82 |
| AEW49 | Southern | 48.45 | 9.48 | *Fagus sylvatica* | 6.31 |
| HEW01 | Central | 51.19 | 10.32 | *Picea abies* | 6.23 |
| HEW02 | Central | 51.21 | 10.37 | *Picea abies* | 6.59 |
| HEW03 | Central | 51.27 | 10.31 | *Picea abies* | 5.07 |
| HEW04 | Central | 51.37 | 10.53 | *Fagus sylvatica* | 6.14 |
| HEW05 | Central | 51.26 | 10.24 | *Fagus sylvatica* | 5.32 |
| HEW06 | Central | 51.27 | 10.24 | *Fagus sylvatica* | 4.35 |
| HEW10 | Central | 51.09 | 10.46 | *Fagus sylvatica* | 4.93 |
| HEW11 | Central | 51.1 | 10.4 | *Fagus sylvatica* | 4.87 |
| HEW12 | Central | 51.1 | 10.46 | *Fagus sylvatica* | 4.14 |
| HEW13 | Central | 51.24 | 10.31 | *Picea abies* | 6.76 |
| HEW16 | Central | 51.18 | 10.37 | *Fagus sylvatica* | 4.86 |
| HEW17 | Central | 51.28 | 10.23 | *Fagus sylvatica* | 3.87 |
| HEW21 | Central | 51.19 | 10.32 | *Fagus sylvatica* | 6.29 |
| HEW22 | Central | 51.34 | 10.36 | *Fagus sylvatica* | 4.82 |
| HEW36 | Central | 51.11 | 10.41 | *Fagus sylvatica* | 4.71 |
| HEW47 | Central | 51.18 | 10.38 | *Fagus sylvatica* | 4.86 |
| SEW01 | Northern | 52.9 | 13.85 | *Pinus sylvestris* | 3.64 |
| SEW02 | Northern | 52.95 | 13.78 | *Pinus sylvestris* | 3.52 |
| SEW03 | Northern | 52.92 | 13.64 | *Pinus sylvestris* | 3.44 |
| SEW04 | Northern | 52.92 | 13.85 | *Pinus sylvestris* | 3.46 |
| SEW05 | Northern | 53.06 | 13.89 | *Fagus sylvatica* | 3.36 |
| SEW06 | Northern | 52.91 | 13.84 | *Fagus sylvatica* | 3.66 |
| SEW07 | Northern | 53.11 | 13.69 | *Fagus sylvatica* | 3.75 |
| SEW08 | Northern | 53.19 | 13.93 | *Fagus sylvatica* | 3.37 |
| SEW09 | Northern | 53.04 | 13.81 | *Fagus sylvatica* | 3.49 |
| SEW18 | Northern | 52.86 | 13.92 | *Pinus sylvestris* | 3.31 |
| SEW35 | Northern | 52.91 | 13.85 | *Fagus sylvatica* | 3.64 |
| SEW36 | Northern | 52.95 | 13.75 | *Fagus sylvatica* | 3.29 |
| SEW37 | Northern | 52.94 | 13.78 | *Fagus sylvatica* | 3.56 |
| SEW41 | Northern | 52.91 | 13.91 | *Fagus sylvatica* | 3.78 |
| SEW43 | Northern | 52.9 | 13.93 | *Fagus sylvatica* | 3.67 |
| SEW48 | Northern | 53.05 | 13.84 | *Fagus sylvatica* | 3.67 |

Table S2. Number of individuals per taxonomic group and site used to extract essential amino acids. To extract essential amino acids from litter, approximately 15 mg of dry and homogenized leaf litter collected from each site was used.

| **Site** | **Oribatida** | **Mesostigmata** | | **Collembola** | | **Chilopoda** | | **Diplopoda** | | **Isopoda** | | **Lumbricidae** | | **Litter** | |
| --- | --- | --- | --- | --- | --- | --- | --- | --- | --- | --- | --- | --- | --- | --- | --- |
| AEW01 | 250 | 250 | 250 | | 11 | | 0 | | 0 | | 2 | | x | |  |
| AEW02 | 250 | 250 | 250 | | 9 | | 1 | | 4 | | 10 | | x | |  |
| AEW03 | 250 | 250 | 250 | | 28 | | 4 | | 38 | | 1 | | x | |  |
| AEW04 | 150 | 50 | 250 | | 6 | | 1 | | 3 | | 12 | | x | |  |
| AEW05 | 250 | 250 | 250 | | 14 | | 2 | | 56 | | 9 | | x | |  |
| AEW06 | 250 | 250 | 250 | | 33 | | 1 | | 6 | | 7 | | x | |  |
| AEW07 | 250 | 250 | 250 | | 31 | | 0 | | 14 | | 3 | | x | |  |
| AEW08 | 250 | 250 | 250 | | 37 | | 2 | | 17 | | 10 | | x | |  |
| AEW09 | 250 | 250 | 250 | | 9 | | 5 | | 16 | | 8 | | x | |  |
| AEW11 | 250 | 250 | 250 | | 11 | | 1 | | 1 | | 0 | | x | |  |
| AEW17 | 140 | 250 | 250 | | 19 | | 9 | | 1 | | 6 | | x | |  |
| AEW18 | 250 | 240 | 250 | | 19 | | 3 | | 6 | | 5 | | x | |  |
| AEW25 | 150 | 80 | 140 | | 7 | | 0 | | 8 | | 11 | | x | |  |
| AEW27 | 215 | 215 | 250 | | 11 | | 4 | | 10 | | 10 | | x | |  |
| AEW30 | 250 | 120 | 250 | | 9 | | 1 | | 4 | | 23 | | x | |  |
| AEW49 | 160 | 50 | 250 | | 9 | | 1 | | 0 | | 15 | | x | |  |
| HEW01 | 150 | 100 | 150 | | 11 | | 4 | | 16 | | 34 | | x | |  |
| HEW02 | 150 | 140 | 150 | | 13 | | 3 | | 0 | | 25 | | x | |  |
| HEW03 | 150 | 150 | 150 | | 15 | | 11 | | 0 | | 16 | | x | |  |
| HEW04 | 150 | 135 | 150 | | 4 | | 8 | | 2 | | 38 | | x | |  |
| HEW05 | 200 | 200 | 200 | | 10 | | 1 | | 11 | | 8 | | x | |  |
| HEW06 | 200 | 200 | 200 | | 20 | | 1 | | 0 | | 24 | | x | |  |
| HEW10 | 250 | 50 | 175 | | 1 | | 1 | | 0 | | 19 | | x | |  |
| HEW11 | 230 | 100 | 250 | | 11 | | 1 | | 0 | | 18 | | x | |  |
| HEW12 | 250 | 250 | 250 | | 10 | | 0 | | 0 | | 17 | | x | |  |
| HEW13 | 250 | 58 | 250 | | 8 | | 2 | | 0 | | 17 | | x | |  |
| HEW16 | 250 | 225 | 250 | | 6 | | 2 | | 2 | | 6 | | x | |  |
| HEW17 | 250 | 250 | 250 | | 3 | | 3 | | 0 | | 9 | | x | |  |
| HEW21 | 250 | 240 | 250 | | 12 | | 3 | | 26 | | 8 | | x | |  |
| HEW22 | 250 | 250 | 250 | | 13 | | 2 | | 1 | | 5 | | x | |  |
| HEW36 | 230 | 165 | 250 | | 1 | | 2 | | 4 | | 12 | | x | |  |
| HEW47 | 250 | 240 | 250 | | 15 | | 4 | | 6 | | 5 | | x | |  |
| SEW01 | 200 | 150 | 140 | | 1 | | 0 | | 1 | | 1 | | x | |  |
| SEW02 | 250 | 250 | 250 | | 5 | | 2 | | 5 | | 0 | | x | |  |
| SEW03 | 100 | 40 | 4 | | 5 | | 0 | | 1 | | 1 | | x | |  |
| SEW04 | 180 | 250 | 115 | | 3 | | 1 | | 0 | | 1 | | x | |  |
| SEW05 | 220 | 250 | 250 | | 0 | | 0 | | 0 | | 0 | | x | |  |
| SEW06 | 250 | 250 | 55 | | 22 | | 0 | | 4 | | 8 | | x | |  |
| SEW07 | 250 | 250 | 215 | | 5 | | 1 | | 0 | | 2 | | x | |  |
| SEW08 | 250 | 250 | 250 | | 42 | | 0 | | 0 | | 14 | | x | |  |
| SEW09 | 230 | 210 | 240 | | 0 | | 2 | | 0 | | 2 | | x | |  |
| SEW18 | 250 | 235 | 250 | | 6 | | 1 | | 0 | | 0 | | x | |  |
| SEW35 | 95 | 250 | 175 | | 16 | | 0 | | 0 | | 1 | | x | |  |
| SEW36 | 250 | 250 | 250 | | 23 | | 0 | | 0 | | 1 | | x | |  |
| SEW37 | 250 | 200 | 250 | | 30 | | 0 | | 0 | | 9 | | x | |  |
| SEW41 | 250 | 225 | 250 | | 33 | | 3 | | 0 | | 2 | | x | |  |
| SEW43 | 250 | 235 | 250 | | 6 | | 1 | | 1 | | 8 | | x | |  |
| SEW48 | 100 | 250 | 150 | | 0 | | 0 | | 0 | | 1 | | x | |  |
|  |  |  |  | |  | |  | |  | |  | |  | |  |
| **N samples** | **48** | **48** | **48** | | **45** | | **36** | | **27** | | **44** | | **48** | |  |

Table S3. Mean and standard deviations (sd) of measured δ^13^C values in a standard mixture of amino acids (AA), derivatized alongside batches of ~ 20 samples. Standards were injected into GC-C-IRMS in triplicates, approximately following four sample measurements. Total mean and standard deviations across standards (total) are given to check reproducibility of GC-C-IRMS measurements. Essential amino acids used in the analysis are indicated with asterisks.

| **AA** | **Std 4** |  | **Std 7** |  | **Std 9** |  | **Std 10** |  | **Std 11** |  | **total** |  |
| --- | --- | --- | --- | --- | --- | --- | --- | --- | --- | --- | --- | --- |
|  | δ^13^C | sd | δ^13^C | sd | δ^13^C | sd | δ^13^C | sd | δ^13^C | sd | mean | sd |
| Ala | -37.43 | 1.46 | -36.26 | 0.20 | -36.43 | 0.49 | -36.66 | 0.20 | -37.05 | 0.18 | -36.77 | 0.42 |
| Asx | -39.39 | 1.54 | -38.99 | 0.27 | -38.31 | 0.05 | -39.04 | 0.07 | -38.09 | 0.23 | -38.76 | 0.49 |
| Glx | -38.30 | 1.13 | -38.80 | 0.49 | -38.07 | 0.25 | -38.56 | 0.04 | -37.80 | 0.40 | -38.31 | 0.35 |
| Ile* | -33.64 | 2.32 | -30.04 | 0.60 | -31.50 | 0.22 | -31.43 | 0.34 | -31.96 | 0.62 | -31.71 | 1.16 |
| Leu* | -41.15 | 0.95 | -41.15 | 0.37 | -41.48 | 0.54 | -41.43 | 0.27 | -41.32 | 0.06 | -41.31 | 0.14 |
| Met | -41.78 | 2.24 | -38.94 | 0.37 | -39.23 | 0.26 | -40.71 | 0.11 | -40.52 | 0.27 | -40.24 | 1.04 |
| Nle | -39.78 | 1.21 | -39.15 | 0.26 | -40.05 | 0.28 | -39.79 | 0.27 | -39.77 | 0.45 | -39.71 | 0.30 |
| Phe* | -25.34 | 1.07 | -24.58 | 0.09 | -24.70 | 0.55 | -25.03 | 0.37 | -24.42 | 0.59 | -24.81 | 0.33 |
| Pro | -30.65 | 2.08 | - | - | - | - | -28.18 | 0.14 | - | - | -29.42 | 1.23 |
| Ser | -38.71 | 1.58 | -37.07 | 0.31 | -37.54 | 0.10 | -37.38 | 0.11 | -37.66 | 0.36 | -37.67 | 0.55 |
| Thr* | -37.92 | 1.05 | -36.83 | 0.32 | -36.97 | 0.29 | -37.40 | 0.14 | -37.27 | 0.36 | -37.28 | 0.38 |
| Tyr | -38.28 | 0.78 | - | - | - | - | -38.33 | 0.16 | - | - | -38.30 | 0.02 |
| Val* | -31.78 | 1.71 | -30.10 | 0.22 | -30.97 | 0.23 | -30.80 | 0.18 | -31.08 | 0.55 | -30.95 | 0.54 |

Table S4. Group-specific length and width mass regressions (length, L, and width, W [mm]) to estimate dry weight (DW) and fresh weight (FW) in µg for soil animal groups with group-specific coefficients (a, b). Dry weights were converted to fresh weights following Mercer et al. (2001). LC is the coefficient for conversion of size units.

| **Animal group** | **Measures** | **LC** | **Group** | **Formula** | **Reference** | **a** | | **b1** | **b2** | **c** | **d** |
| --- | --- | --- | --- | --- | --- | --- | --- | --- | --- | --- | --- |
| **Annelida** | L | 1 | Annelida (here: Lumbricidae) | DW[µg]=a*(L[mm]*Coeff)^b1; FW[µg]=c*DW^d | Hale et al (2004); Potapov et al. (2019) | 6.8 | 2.2853 | | - | 8.48 | 1.09 |
| **Chilopoda1** | LW | 1 | Chilopoda  (Lithobiomorpha and Scolopendromorpha) | DW[µg]=a*(L[mm]*Coeff)^b1*(W[mm]*Coeff)^b2; FW[µg]=c*DW^d | Sohlström et al. (2018) | 44.67 | 2.112 | | 0.742 | 1 | 1 |
| **Chilopoda2** | LW | 1 | Chilopoda (Geophilomorpha) | DW[µg]=a*(L[mm]*Coeff)^b1*(W[mm]*Coeff)^b2; FW[µg]=c*DW^d | Sohlström et al. (2018) | 380.2 | 0.964 | | 1.766 | 1 | 1 |
| **Diplopoda** | LW | 1 | Diplopoda | DW[µg]=a*(L[mm]*Coeff)^b1*(W[mm]*Coeff)^b2; FW[µg]=c*DW^d | Sohlström et al. (2018) | 662.2 | 1.012 | | 2.191 | 1 | 1 |
| **Isopoda** | LW | 1 | Isopoda | DW[µg]=a*(L[mm]*Coeff)^b1*(W[mm]*Coeff)^b2; FW[µg]=c*DW^d | Sohlström et al. (2018) | 158.5 | 1.646 | | 1.154 | 1 | 1 |
| **Collembola1** | L | 1 | Collembola (Arthopleona) | DW[µg]=a*(L[mm]*Coeff)^b1; FW[µg]=c*DW^d; | Petersen (1975) | 5.6 | 2.69254546 | | - | 4.08 | 1.02 |
| **Collembola2** | L | 0.83 | Collembola (Symphypleona) | DW[µg]=a*(L[mm]*Coeff)^b1; FW[µg]=c*DW^d; | Petersen (1975) | 57.8 | 2.954 | | - | 4.08 | 1.02 |
| **Oribatida** | L | 1000 | Oribatida (juveniles) | DW[µg]=a*(L[mm]*Coeff)^b1; FW[µg]=c*DW^d | Lu et al. (2024) | 7.48E-08 | 3.093 | | - | 1 | 1 |
| **Mesostigmata** | LW | 1000 | Mesostigmata | DW[µg]=a*(L[mm]*Coeff)^b1*(W[mm]*Coeff )^b2; FW[µg]=c*DW^d | Newton and Proctor (2013) | 2.14E-07 | 1.53 | | 1.53 | 1 | 1 |

|  |  |  | **δ^13^C values of eAAs** | | |  | **Trophic position** | | |
| --- | --- | --- | --- | --- | --- | --- | --- | --- | --- |
| Animal group | *n* | Factor | Df | F | p | *n* | Df | F | p |
| All animal groups |  | **Animal group** | **6** | **26.21** | **< 0.001** |  | **6** | **191.22** | **< 0.001** |
|  |  | **Forest type** | **3** | **4.16** | **< 0.001** |  | 3 | 1.69 | 0.16 |
|  |  | **Region** | **2** | **3.18** | **0.013** |  | **2** | **9.25** | **< 0.001** |
|  |  | Animal group × forest type | 18 | 1.16 | 0.242 |  | 18 | 1.08 | 0.366 |
|  |  | **Animal group × region** | **12** | **2.44** | **< 0.001** |  | **12** | **2.32** | **0.008** |
|  |  | Forest type × region | 6 | 0.65 | 0.794 |  | 6 | 0.743 | 0.615 |
|  |  | Animal Group × forest type × region | 32 | 1.03 | 0.408 |  | 32 | 0.743 | 0.838 |
|  |  | Residuals | 196 |  |  |  | 177 |  |  |
|  | *269* |  |  |  |  | *257* |  |  |  |
| Lumbricidae |  | Forest type | 3 | 1.24 | 0.295 |  | 3 | 1.66 | 0.194 |
|  |  | **Region** | **2** | **5.10** | **0.001** |  | **2** | **3.53** | **0.041** |
|  |  | Forest type × region | 6 | 0.46 | 0.928 |  | 6 | 0.25 | 0.955 |
|  |  | Residuals | 32 |  |  |  | 31 |  |  |
|  | *44* |  |  |  |  | *43* |  |  |  |
| Oribatida |  | Forest type | 3 | 2.02 | 0.072 |  | 3 | 1.06 | 0.375 |
|  |  | **Region** | **2** | **4.02** | **0.005** |  | **2** | **7.55** | **0.002** |
|  |  | **Forest type × region** | **6** | **2.11** | **0.026** |  | 6 | 0.69 | 0.652 |
|  |  | Residuals | 36 |  |  |  | 35 |  |  |
|  | *48* |  |  |  |  | *47* |  |  |  |
| Diplopoda |  | Forest type | 3 | 1.41 | 0.227 |  | 3 | 0.72 | 0.553 |
|  |  | **Region** | **2** | **3.01** | **0.027** |  | 2 | 3.48 | 0.054 |
|  |  | Forest type × region | 5 | 0.58 | 0.818 |  | 5 | 0.69 | 0.636 |
|  |  | Residuals | 24 |  |  |  | 17 |  |  |
|  | *35* |  |  |  |  | *28* |  |  |  |
| Collembola |  | Forest type | 3 | 1.53 | 0.181 |  | 3 | 0.18 | 0.909 |
|  |  | Region | 2 | 0.43 | 0.781 |  | **2** | **3.86** | **0.032** |
|  |  | Forest type × region | 6 | 0.88 | 0.569 |  | 6 | 0.86 | 0.537 |
|  |  | Residuals | 32 |  |  |  | 30 |  |  |
|  | *44* |  |  |  |  | *42* |  |  |  |
| Chilopoda |  | Forest type | 3 | 1.83 | 0.108 |  | 3 | 1.10 | 0.361 |
|  |  | Region | 2 | 1.83 | 0.134 |  | 2 | 2.16 | 0.131 |
|  |  | Forest type × region | 6 | 1.35 | 0.213 |  | 6 | 1.77 | 0.136 |
|  |  | Residuals | 30 |  |  |  | 32 |  |  |
|  | *42* |  |  |  |  | *44* |  |  |  |
| Isopoda |  | Forest type | 3 | 0.64 | 0.694 |  | 3 | 0.33 | 0.8 |
|  |  | Region | 2 | 0.28 | 0.883 |  | 2 | 0.10 | 0.90 |
|  |  | Forest type × region | 3 | 0.53 | 0.772 |  | 3 | 0.03 | 0.99 |
|  |  | Residuals | 8 |  |  |  | 2 |  |  |
|  | *17* |  |  |  |  | *11* |  |  |  |
| Mesostigmata | | Forest type | 3 | 1.44 | 0.209 |  | 3 | 2.08 | 0.124 |
|  |  | Region | **2** | **4.85** | **0.001** |  | **2** | **3.71** | **0.036** |
|  |  | Forest type × region | 6 | 1.20 | 0.298 |  | 6 | 0.90 | 0.506 |
|  |  | Residuals | 34 |  |  |  | 30 |  |  |
|  | *46* |  |  |  | *48* | *42* |  |  |  |

Table S5. Manova and linear model statistics and for the effect of Animal group, Forest type, Region and their interaction on linear discriminant scores of LD1 and LD2 (left) and trophic positions (right) of soil animal communities as response variables, respectively. Significant effects (p< 0.05) are highlighted in bold.

| Table S6. Anova statistics for the effect of Animal group, Forest type, Region and Basal resource (fungi, plants and bacteria) and their interaction on energy fluxes in soil animal communities (Animal group). Energy fluxes used as response variables were calculated by multiplying the energy intake with the relative contribution of fungi, plants, and bacteria estimated in Bayesian mixing models for all groups in either forest types (left) or regions (right) (see Fig. S3b,c). Significant effects (p< 0.05) are highlighted in bold. | | | | | | | | |
| --- | --- | --- | --- | --- | --- | --- | --- | --- |
| Animal group | Factor | Df | F | p | Factor | Df | F | p |
| All animal groups | **Animal group** | **6** | **308.25** | **< 0.001** | **Animal group** | **6** | **202.18** | **< 0.001** |
|  | Forest type | 3 | 1.72 | 0.161 | **Region** | **2** | **46.28** | **< 0.001** |
|  | **Basal Resource** | **2** | **451.56** | **< 0.001** | **Resource** | **2** | **308.37** | **< 0.001** |
|  | **Animal group × forest type** | **18** | **2.21** | **0.003** | **Animal group × Region** | **12** | **7.05** | **< 0.001** |
|  | **Animal group × Basal resource** | **12** | **18.69** | **< 0.001** | **Animal group × Basal resource** | **12** | **19.68** | **< 0.001** |
|  | Forest type × Basal resource | 6 | 1.47 | 0.183 | Region × Basal resource | 4 | 1.76 | 0.13 |
|  | Animal group × forest type × Basal resource | 36 | 0.52 | 0.991 | Animal group × Region × Basal resource | 20 | 0.69 | 0.83 |
|  | Residuals | 924 |  |  | Residuals | 954 |  |  |
|  |  |  |  |  |  |  |  |  |
| Lumbricidae | **Forest type** | **3** | **5.24** | **0.002** | **Region** | **2** | **14.16** | **< 0.001** |
|  | **Resource** | **2** | **114.15** | **< 0.001** | **Resource** | **2** | **63.14** | **< 0.001** |
|  | Forest type × Basal resource | 6 | 0.75 | 0.601 | Region × Basal resource | 4 | 0.54 | 0.702 |
|  | Residuals | 132 |  |  | Residuals | 135 |  |  |
|  |  |  |  |  |  |  |  |  |
| Oribatida | Forest type | 3 | 0.35 | 0.790 | **Region** | **2** | **10.65** | **0.001** |
|  | **Resource** | **2** | **620.90** | **< 0.001** | **Resource** | **2** | **713.60** | **< 0.001** |
|  | Forest type × Basal resource | 6 | 0.90 | 0.496 | **Region × Basal resource** | **4** | **3.20** | **0.015** |
|  | Residuals | 132 |  |  | Residuals | 135 |  |  |
|  |  |  |  |  |  |  |  |  |
| Diplopoda | Forest type | 3 | 1.62 | 0.189 | **Region** | **2** | **11.84** | **< 0.001** |
|  | **Resource** | **2** | **15.83** | **< 0.001** | **Resource** | **2** | **21.74** | **< 0.001** |
|  | Forest type × Basal resource | 6 | 0.906 | 0.492 | Region × Basal resource | 4 | 0.82 | 0.514 |
|  | Residuals | 132 |  |  | Residuals | 135 |  |  |
|  |  |  |  |  |  |  |  |  |
| Collembola | Forest type | 3 | 1.5 | 0.215 | **Region** | **2** | **8.74** | **< 0.001** |
|  | **Resource** | **2** | **251.68** | **< 0.001** | **Resource** | **2** | **325.45** | **< 0.001** |
|  | Forest type × Basal resource | 6 | 0.53 | 0.79 | Region × Basal resource | 4 | 1.73 | 0.147 |
|  | Residuals | 132 |  |  | Residuals | 135 |  |  |
|  |  |  |  |  |  |  |  |  |
| Chilopoda | Forest type | 3 | 2.67 | 0.050 | **Region** | **2** | **4.60** | **0.01** |
|  | **Resource** | **2** | **67.89** | **< 0.001** | **Resource** | **2** | **65.14** | **< 0.001** |
|  | Forest type × Basal resource | 6 | 0.92 | 0.480 | Region × Basal resource | 4 | 0.22 | 0.93 |
|  | Residuals | 132 |  |  | Residuals | 135 |  |  |
|  |  |  |  |  |  |  |  |  |
| Isopoda | Forest type | 3 | 1.40 | 0.246 | **Region** | **2** | **14.163** | **< 0.001** |
|  | **Resource** | **2** | **9.96** | **< 0.001** | **Resource** | **2** | **11.40** | **< 0.001** |
|  | Forest type × Basal resource | 6 | 0.20 | 0.975 | Region × Basal resource | 4 | 0.69 | 0.147 |
|  | Residuals | 132 |  |  | Residuals | 135 |  |  |
|  |  |  |  |  |  |  |  |  |
| Mesostigmata | Forest type | 3 | 0.59 | 0.620 | **Region** | **2** | **8.08** | **< 0.001** |
|  | **Resource** | **2** | **186.78** | **< 0.001** | **Resource** | **2** | **402.27** | **< 0.001** |
|  | **Forest type × Basal resource** | **6** | **2.40** | **0.031** | Region × Basal resource | 4 | 1.63 | 0.170 |
|  | Residuals | 132 |  |  | Residuals | 135 |  |  |
|  |  |  |  |  |  |  |  |  |


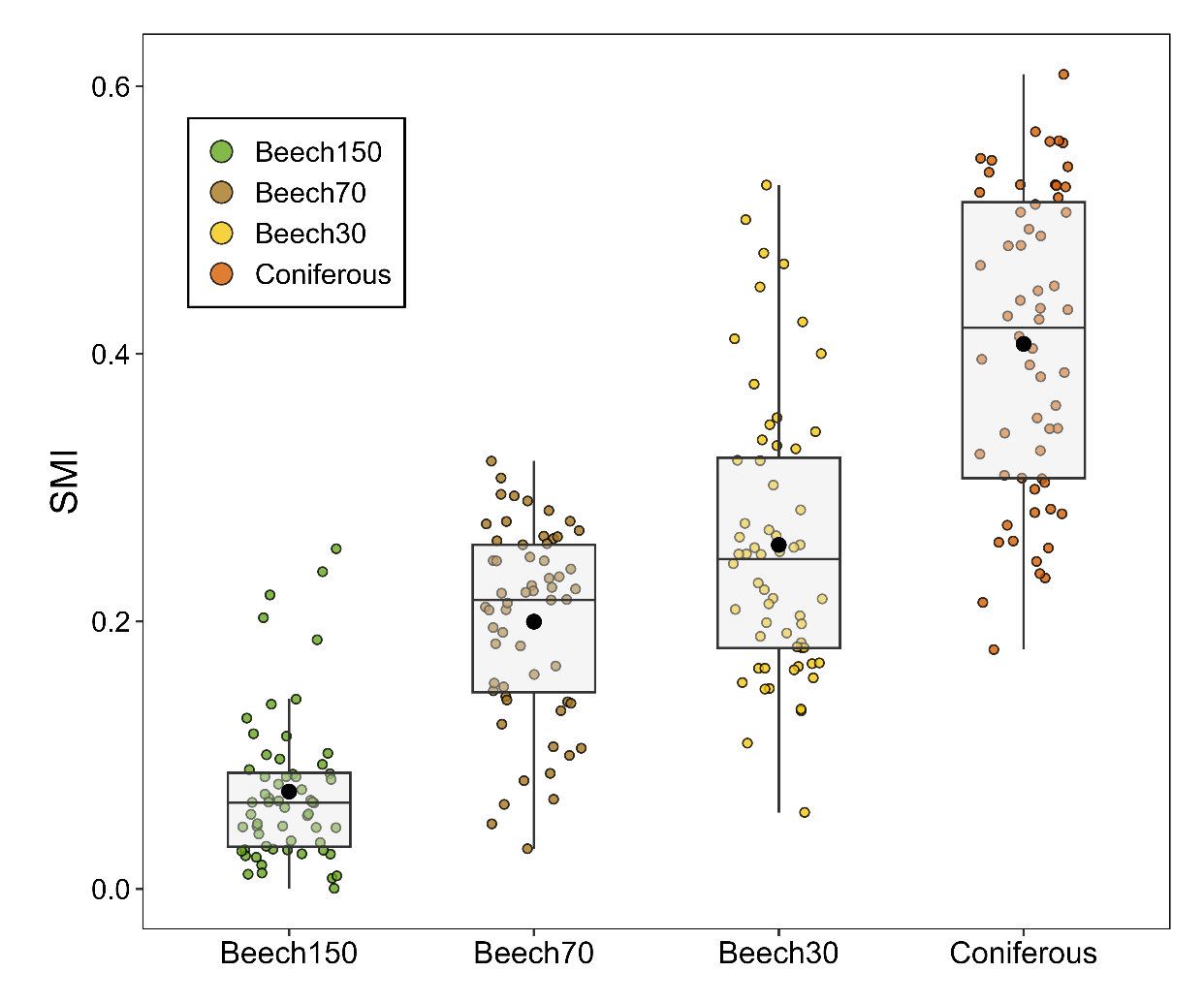


Figure S1. Indicator of silvicultural management intensity (SMI) based on forest inventories conducted in three-year intervals from 2008 to 2020 across three study regions in Germany for unmanaged beech forests (Beech150), old managed beech forests (Beech70), young managed beech forests (Beech30) and coniferous forests (Coniferous) calculated from stand age, proportion of non-native tree species and the deviation of the actual stocking from a fully stocked mature forest due to harvesting and thinning (Schall & Ammer 2013; 2023); black circles represent means, horizontal lines medians and boxes the interquartile range. The linear model with forest type as fixed factor resulted in an adjusted R^2^ of 0.64.


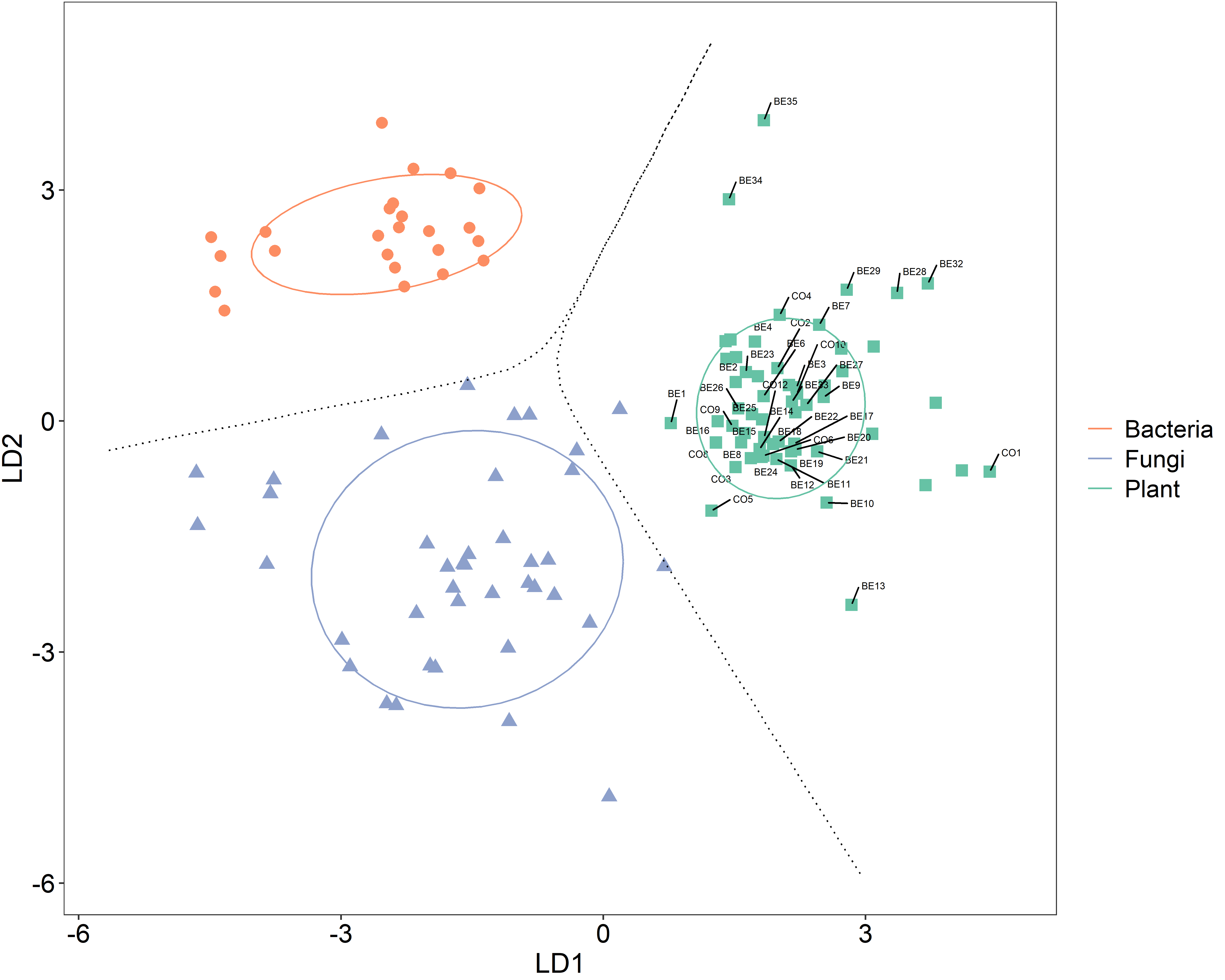


Figure S2. Linear discriminant analysis (LDA) of δ^13^C values in essential amino acids isoleucine, leucine, phenylalanine, threonine and valine of training data from bacteria (light orange, n = 26), fungi (light blue, n = 39) and plants (light green, n = 70). Training data was obtained from Larsen (2013, 2016) and Pollierer et al. (2020) and δ^13^C eAA values of litter from beech forests (BE) and coniferous forests (CO) obtained in this study. Ellipses are drawn around linear discriminant means of basal resources and represent 75 % confidence intervals (solid lines). Reclassification success of eAA sources is 99 %.


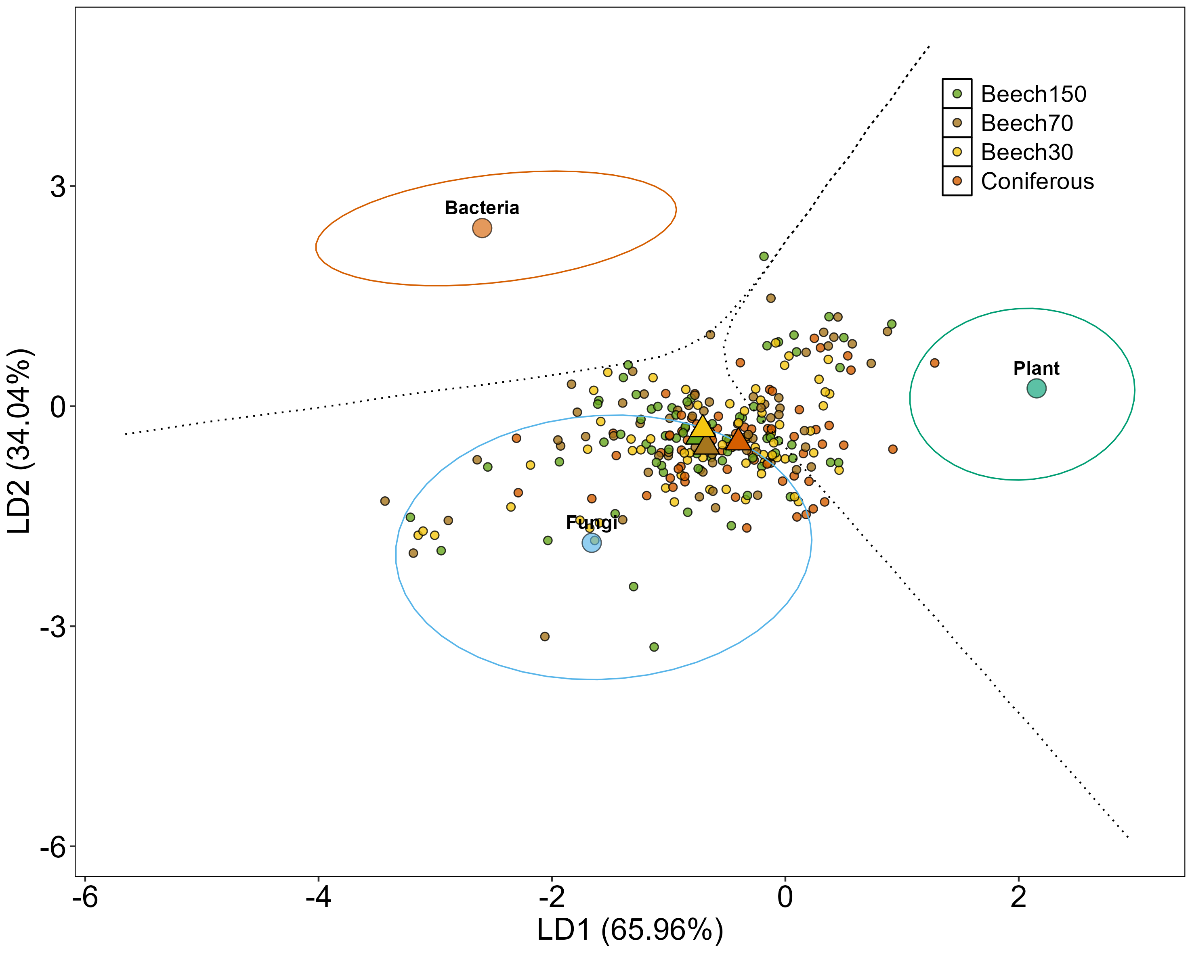


Figure S3. Linear discriminant analysis (LDA) of δ13C values in essential amino acids isoleucine, leucine, phenylalanine, threonine and valine across soil animal communities (Lumbricidae, Oribatida Diplopoda, Collembola, Chilopoda, Isopoda, Mesostigmata) in unmanaged beech forests (Beech150; green), old managed young beech forests (Beech70; brown), young managed beech forests (Beech30; yellow), and coniferous forests (Coniferous; orange). Linear discriminant means for forest types are shown as triangles. Training data used as endmembers to classify δ13C values in eAAs of consumers to their biosynthetic origin come from bacteria (light orange, n = 26), fungi (light blue, n = 39) and plants (light green, n = 70). Ellipses around linear discriminant means of basal resources (points) represent 75 % confidence intervals.


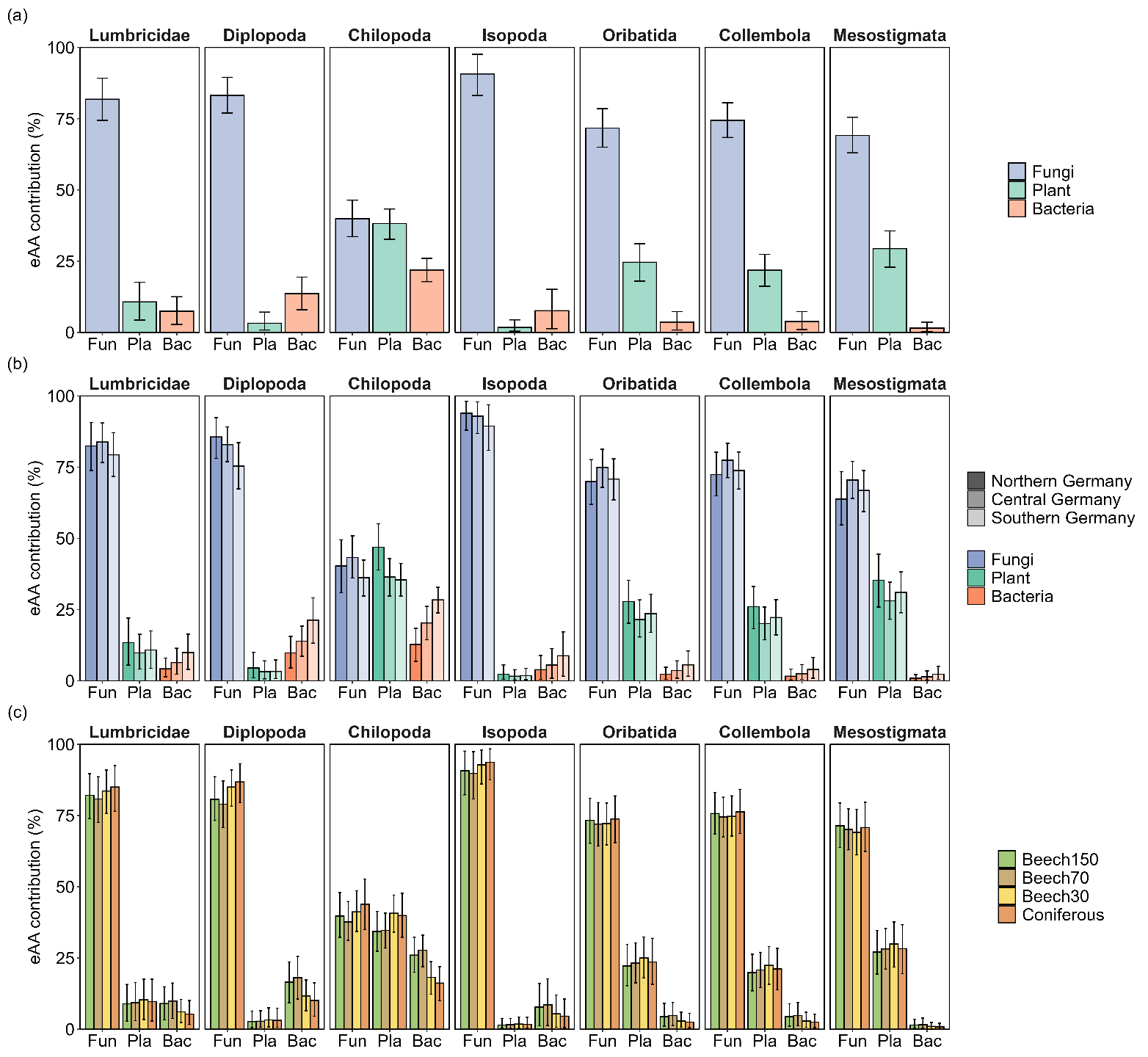


Figure S4. Mean relative contribution (%) ± 95 % credible intervals of essential amino acids (eAA) isoleucine, leucine, phenylalanine, threonine and valine from fungi (Fun), plants (Pla) and bacteria (Bac) to the diet of soil animal groups across forest types of different management intensity [unmanaged beech forests (Beech150) old managed beech forests (Beech70), young managed beech forests (Beech30) and coniferous forests (Coniferous)] and across three regions in Germany (Northern Germany, Central Germany, and Southern Germany) (a), in different regions across forest types of different management intensity (b), and in forest types of different management intensity across regions (c).


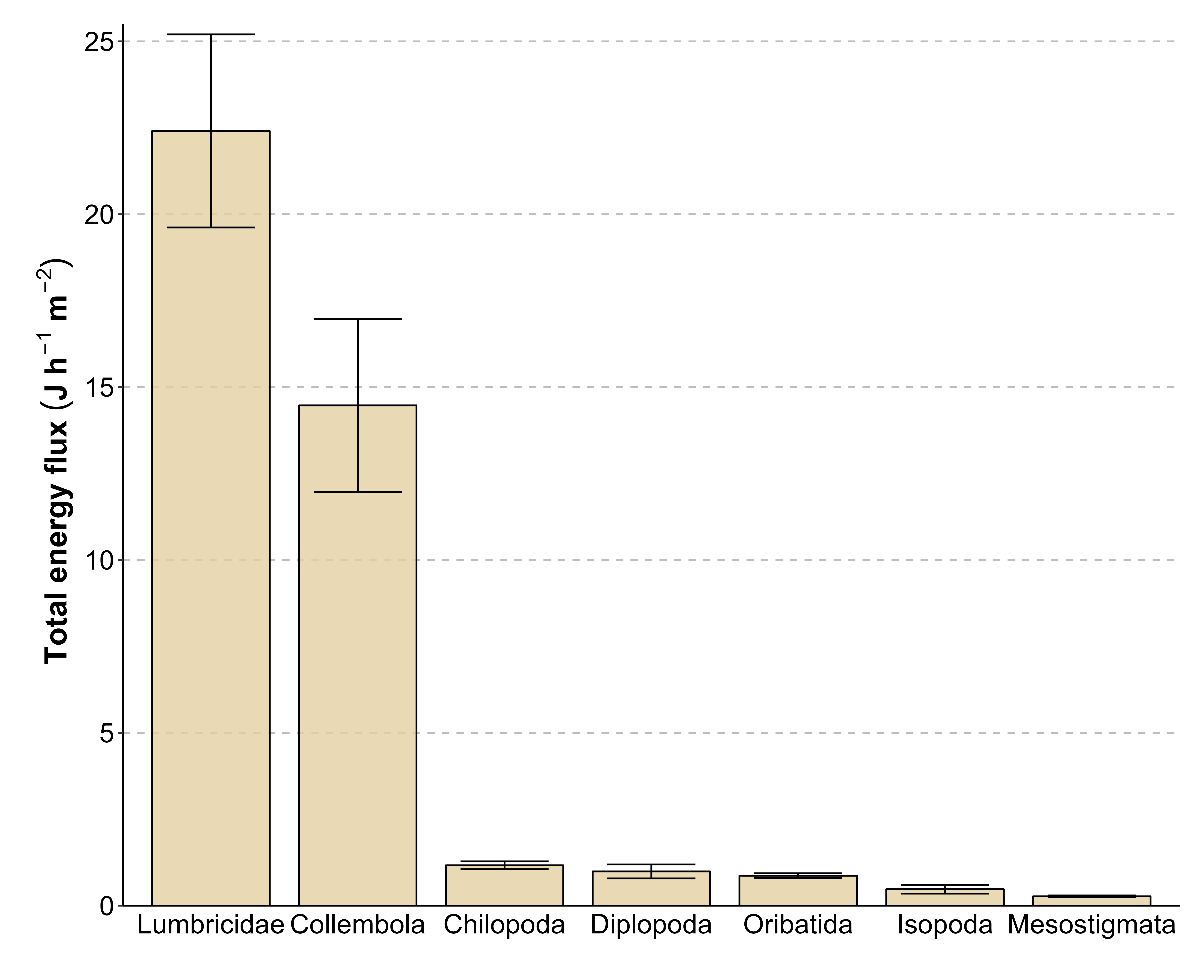


Figure S5. Mean total energy fluxes (J h^-1^ m^-2^ ± SE) calculated as the sum of energy fluxes from basal resources of fungi, plants and bacteria to soil animal communities across forest types of different management intensity and three regions in Germany.


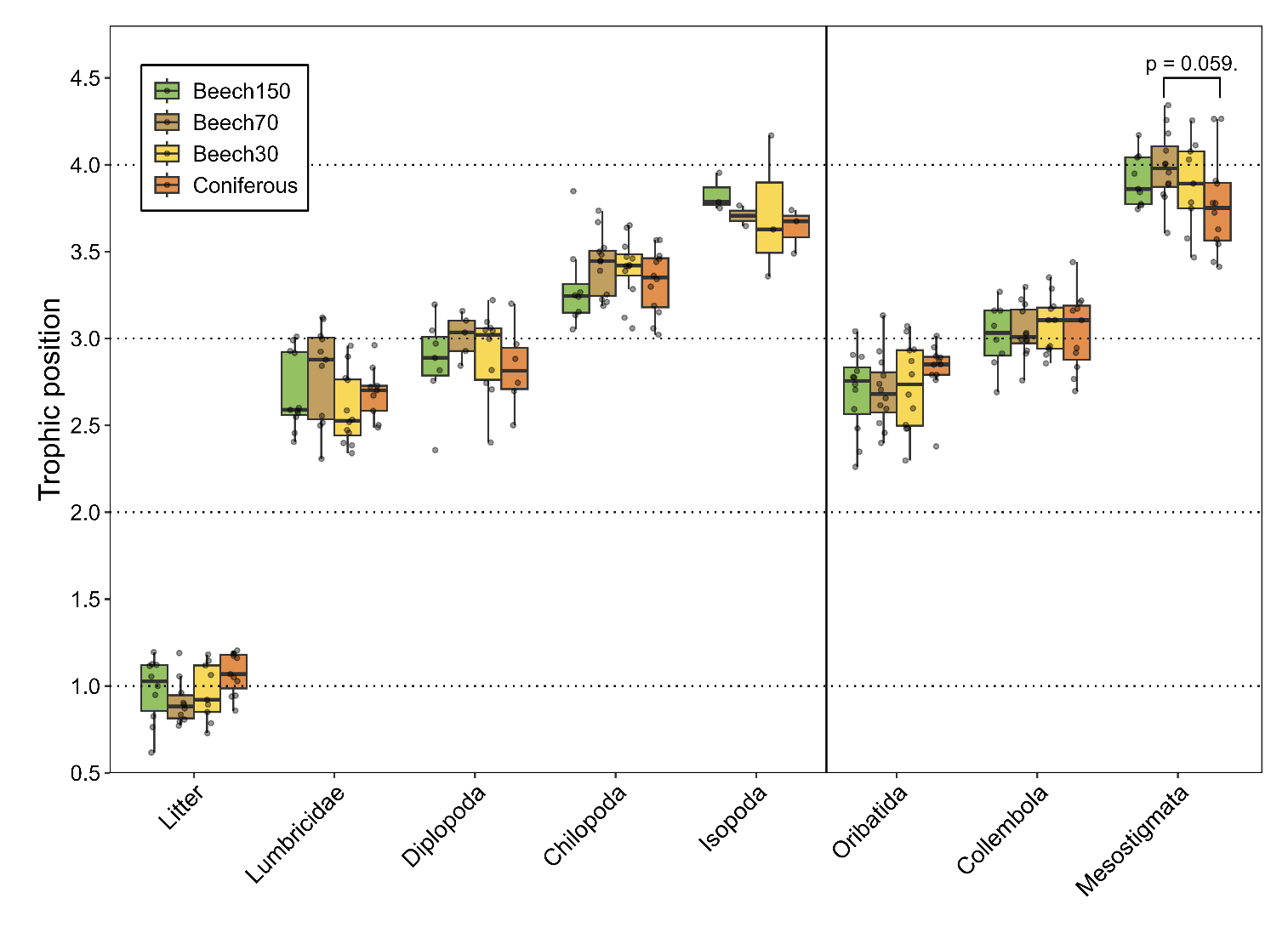


Figure S6 . Trophic position of litter, Lumbricidae, Diplopoda, Chilopoda, Isopoda, Oribatida, Collembola, Mesostigmata in unmanaged beech forests (Beech150), old managed beech forests (Beech70), young managed beech forests (Beech30) and coniferous forests (Coniferous) across northern, central and southern Germany calculated from δ^15^N values of glutamic acid and phenylalanine; black circles represent means, horizontal lines medians and boxes the interquartile range. Trophic position 2 represents consumers feeding on plant resources, such as litter, trophic position 3 indicates feeding on fungi, bacteria or animals of trophic position 2, and trophic position 4 represents predators of microbivores and intraguild predators. Marginally significant (p < 0.10) multiple comparison results (Tukey’s HSD) for TPs of Mesostigmata between Beech70 and Coniferous forests are indicated in the plot.


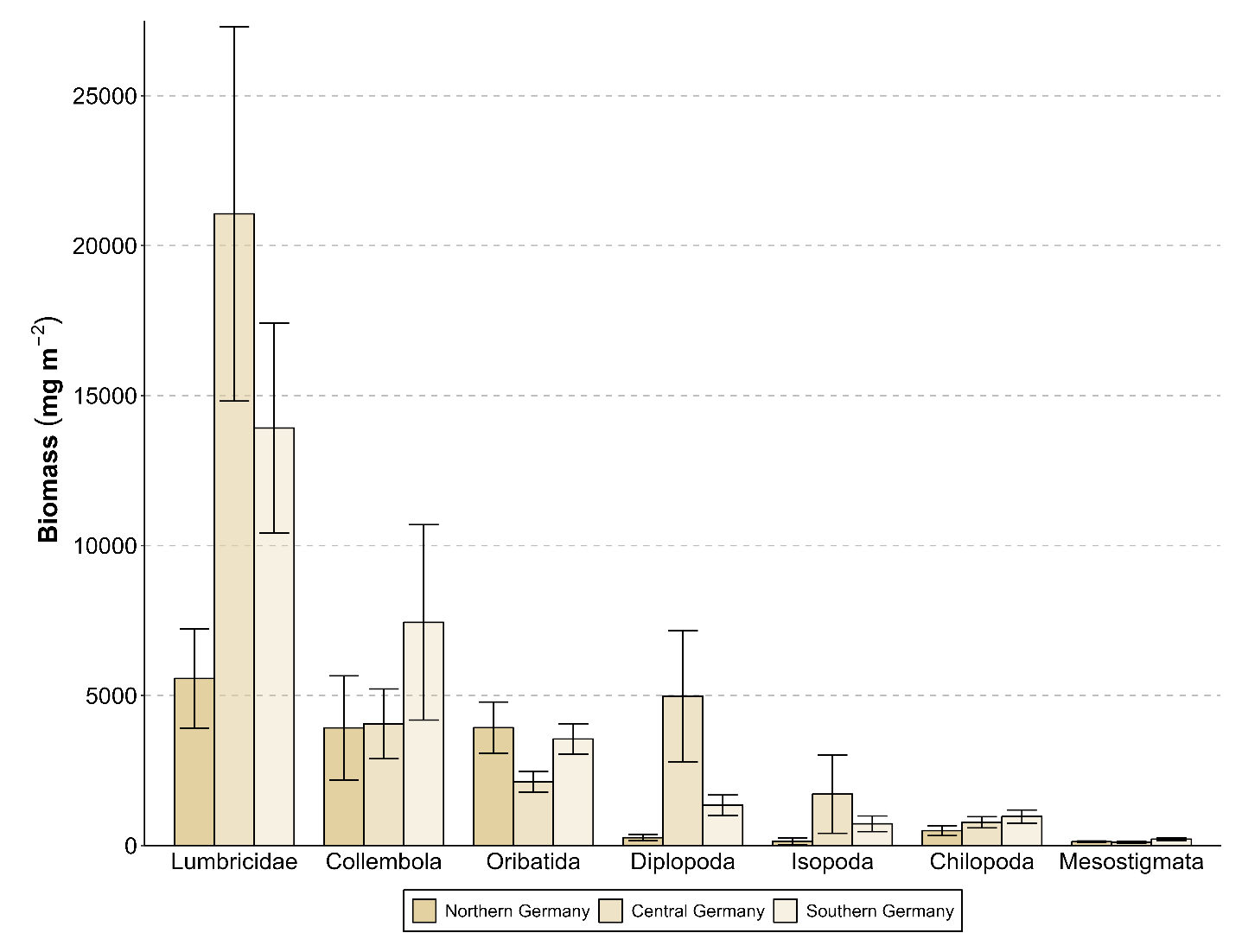


Figure S7. Mean biomass (mg m^-2^ ± SE) for soil animal groups, calculated as the sum of individual fresh masses per site, averaged across forest types in northern, central and southern Germany. Mean biomasses for Mesostigmata were 125.3, 106.5 and 210.5 mg m^-2^ in northern, central and southern Germany, respectively.


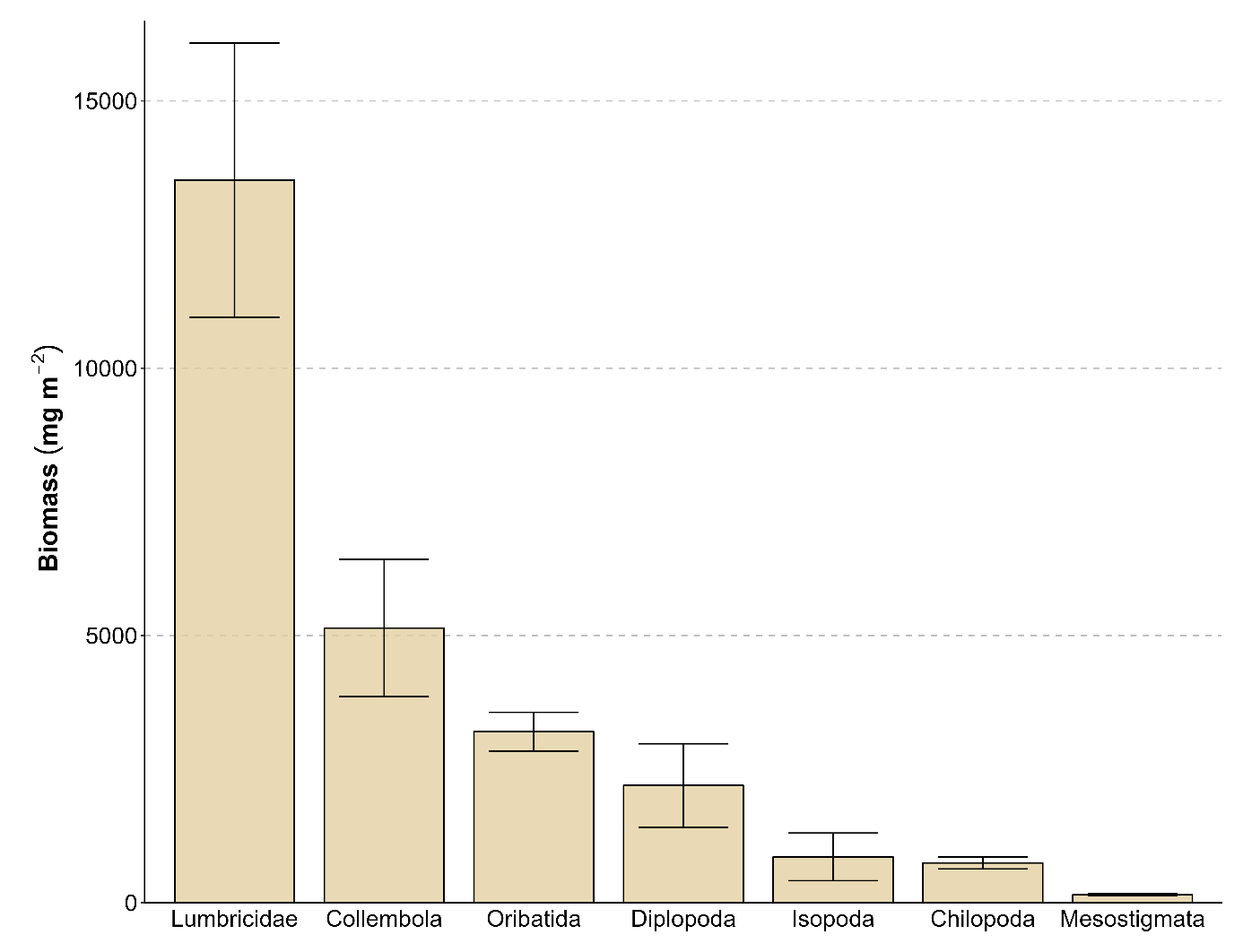


Figure S8. Mean biomass (mg m^-2^ ± SE) for soil animal groups, calculated as the sum of individual fresh masses per site, averaged across forest types and three regions in northern, central and southern Germany. Mean biomass of Mesostigmata was 147.3 mg m^-2^.


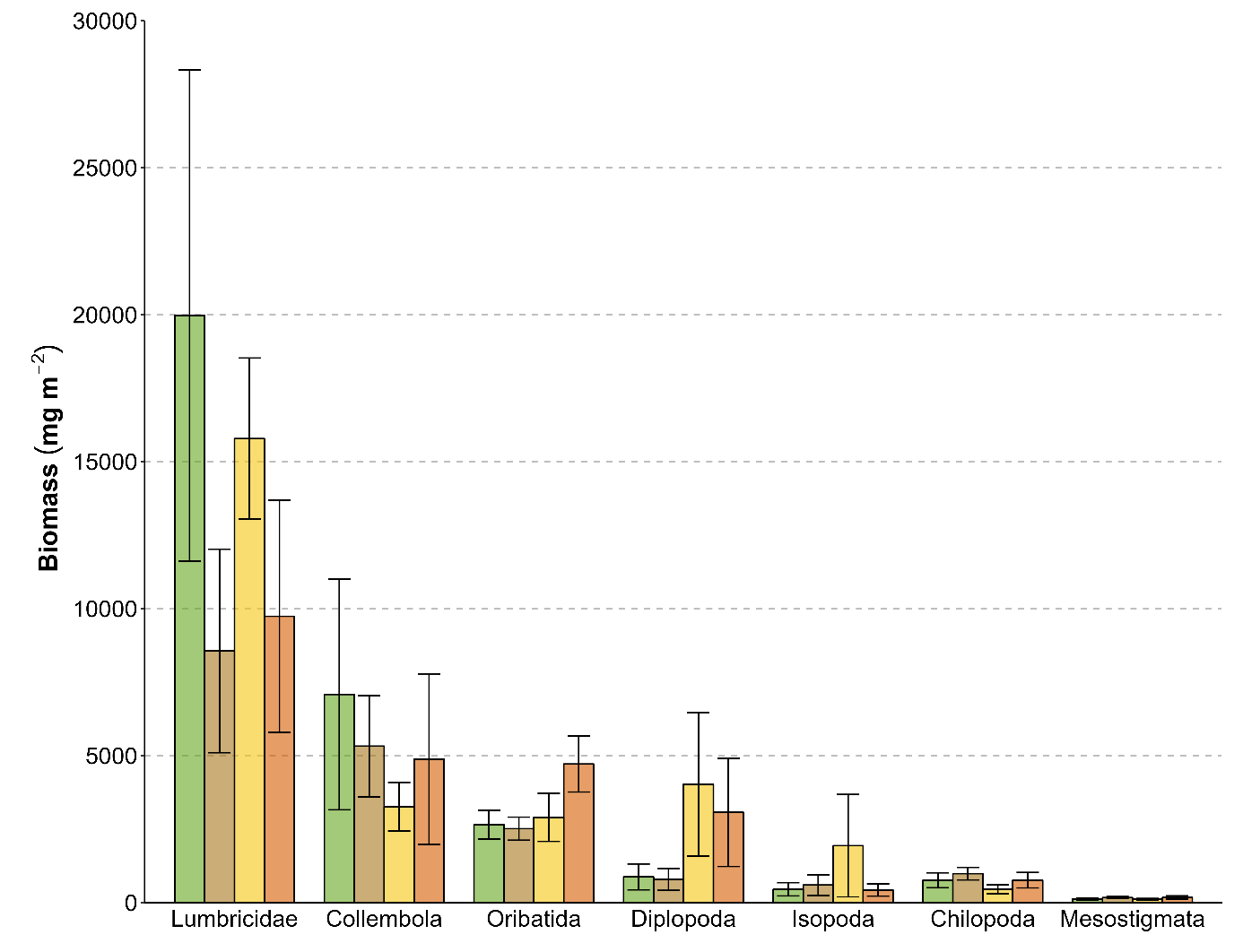


Figure S9. Mean biomass (mg m^-2^ ± SE) for soil animal groups, calculated as the sum of individual fresh masses per site in unmanaged beech forests (green), old managed beech forests (brown), young managed beech forests (yellow) and coniferous forests (orange). Mean biomasses for Mesostigmata were 123.03, 173.4, 116.1, 177.3 mg m^-2^ in unmanaged beech forests, old managed beech forests, young managed beech forests and coniferous forests, respectively.

References

Corr, Lorna T.; Berstan, Robert; Evershed, Richard P. (2007): Development of N-acetyl methyl ester derivatives for the determination of delta13C values of amino acids using gas chromatography-combustion- isotope ratio mass spectrometry. In *Analytical chemistry* 79 (23), pp. 9082–9090. DOI: 10.1021/ac071223b.

Hale, Cindy M. (2004): Allometric Equations for Estimation of Ash-Free Dry Mass from Length Measurements for Selected European Earthworm Species (Lumbricidae) in the Western Great Lakes Region. In *The American Midland Naturalist* 151 (1), pp. 179–185.

Lu, Jing‐Zhong; Wenglein, Ronja; Bluhm, Christian; Stuckenberg, Thalea; Potapov, Anton M.; Ammer, Christian; Scheu, Stefan (2024): Reduced predation and energy flux in soil food webs by introduced tree species: Bottom‐up control of multitrophic biodiversity across size compartments. In *Functional Ecology*, Article 1365-2435.14696. DOI: 10.1111/1365-2435.14696.

Newton, Jeffrey S.; Proctor, Heather C. (2013): A fresh look at weight-estimation models for soil mites (Acari). In *International Journal of Acarology* 39 (1), pp. 72–85. DOI: 10.1080/01647954.2012.744351.

Petersen, Henning (1975): Estimation of dry weight, fresh weight, and calorific content of various Collembolan species. In *Pedobiologia* 15 (3), pp. 222–243.

Potapov, Anton M.; Klarner, Bernhard; Sandmann, Dorothee; Widyastuti, Rahayu; Scheu, Stefan (2019): Linking size spectrum, energy flux and trophic multifunctionality in soil food webs of tropical land-use systems. In *The Journal of animal ecology* 88 (12), pp. 1845–1859. DOI: 10.1111/1365-2656.13027.

Schall, P. & Ammer, C. (2013). How to quantify forest management intensity in Central European forests. *Eur. J. Forest Res.*, 132, 379–396.

Schall, P. & Ammer, C. (2023). SMI annual dynamics - Silvicultural Management Intensity Dynamics on all forest EPs, 2008 - 2020. Version 9. Biodiversity Exploratories Information System. Dataset ID= 31217

Schöning, Ingo (2024): Soil pH - soil sampling campaign 2017, all experimental plots (EPs), 0-10 cm. Version 6. Biodiversity Exploratories Information System. Dataset. https://www.bexis.uni-jena.de/ddm/data/Showdata/22246?version=6

Sohlström, Esra H.; Marian, Lucas; Barnes, Andrew D.; Haneda, Noor F.; Scheu, Stefan; Rall, Björn C. et al. (2018): Applying generalized allometric regressions to predict live body mass of tropical and temperate arthropods. In *Ecology and evolution* 8 (24), pp. 12737–12749. DOI: 10.1002/ece3.4702.
